# Auditory evoked responses during ambulatory OP-MEG

**DOI:** 10.1162/IMAG.a.1424

**Published:** 2026-11-03

**Authors:** Stephanie Mellor, Tim M. Tierney, James Osborne, Meaghan E. Spedden, Robert A. Seymour, Nicholas A. Alexander, Cody Doyle, David Bobela, George C. O’Neill, Katarzyna Rudzka, Sahitya Puvvada, Maike Schmidt, Vishal Shah, Gareth R. Barnes

**Affiliations:** Department of Imaging Neuroscience, UCL Queen Square Institute of Neurology, University College London, London, United Kingdom; Spinal Cord Injury Center, Balgrist University Hospital, University of Zurich, Zurich, Switzerland; Translational Neuromodeling Unit, Institute for Biomedical Engineering, University of Zurich & ETH Zurich, Zurich, Switzerland; QuSpin Inc., Louisville, CO, United States; Oxford Centre for Human Brain Activity (OHBA), Department of Psychiatry, University of Oxford, Oxford, United Kingdom; Department of Neuroscience, Physiology and Pharmacology, University College London, London, United Kingdom; UCL Institute of Cognitive Neuroscience, University College London, London, United Kingdom

**Keywords:** magnetoencephalography, optically pumped magnetometer, ambulatory OP-MEG, auditory evoked response, mismatch negativity

## Abstract

The latest generation of optically pumped magnetometer (OPM)-based magnetoencephalography (OP-MEG) systems are increasingly portable and lightweight, making the system easier to wear while undertaking large, whole-body translational and rotational movements. We recorded OP-MEG from three participants during auditory stimulation, while the participants walked around a magnetically shielded room, moving across an area of at least 1.7 m^2^ and rotating by at least 180 degrees. All sensor-specific electronics were housed within a wearable backpack, minimising movement restrictions which would otherwise be imposed by sensor cabling. In this experiment, the maximum peak-to-peak range of the observed magnetic field recorded on a single channel was 25.2 nT, considerably beyond the approximately 4 nT range of many previous OP-MEG systems. We show that adaptive multipole modelling (AMM) with the temporal extension can be used to separate the interference and neuromagnetic signals, despite the large degree of movement and proximity of the sensor electronics. We observed significant auditory evoked responses at the sensor level, without the need for source localisation approaches for interference suppression. These findings demonstrate the capability of OP-MEG for conducting naturalistic experiments involving movement and for recordings with paediatric and clinical cohorts who cannot remain still.

## 1 Introduction

Magnetoencephalography (MEG) is a non-invasive neuroimaging modality, where extracranial magnetic fields due to neuronal current flow are measured (Hämäläinen et al., 1993). These measurements are generally undertaken with cryogenically cooled sensors. However, non-cryogenic sensors, most prominently optically pumped magnetometers (OPMs), have become viable alternatives comparatively recently (Sander et al., 2012; Shah & Wakai, 2013). OPMs can be placed directly on the scalp to measure these magnetic fields, and worn as the participant moves their head (Boto et al., 2018; Seymour et al., 2021). The development of OPM-based MEG (OP-MEG) has, therefore, led to increased interest in neuroimaging during movement and naturalistic behaviour (Hill et al., 2019; O’Neill et al., 2025; Roberts et al., 2019).

While the self-contained nature of OPMs makes large movements during OP-MEG feasible, in practice, movement in OP-MEG has been limited by background magnetic field changes. Not only do changes in position and orientation introduce magnetic interference, but also many OPMs have an intrinsic trade-off between sensitivity and operating range (Knappe et al., 2016; Tierney et al., 2019). Early iterations of commercial OPMs using rubidium vapour were highly sensitive but linearity decreased quickly beyond approximately 2 nT (Iivanainen et al., 2019; Osborne et al., 2018; Tierney et al., 2019). Additionally, measurements can be affected by magnetic field changes in orthogonal directions to the recording axis (Borna et al., 2022). To minimise background interference, MEG is typically undertaken within a magnetically shielded room (MSR) and additional active magnetic shielding may be used to further reduce the background field (Holmes, Rea, et al., 2023; Holmes et al., 2018; Iivanainen et al., 2019). Nevertheless, within the MSR at UCL for example, despite high performance at the centre of the room (<2 nT static field), the magnetic field quickly increases outside of the central cubic meter (Mellor et al., 2022).

The Neuro-1 system (QuSpin, CO) is a new OP-MEG system where all of the individual sensor electronics are connected within a backpack which can be worn by the participant as they move (Schofield et al., 2024). Individual sensor wires pass from the OPMs to the backpack, but only two cables connect from the backpack to outside of the MSR. The reduced cabling passing outside of the MSR means that participants can more easily move around the MSR than with earlier versions of these OPM sensors (Spedden et al., 2025), although the limitations from interference from the ambient magnetic field remain. Additionally, the backpack itself provides a source of interference proximal to the OPMs.

Assuming that the ambient magnetic field is low enough that the OPMs are within their operating range, the signal-to-noise ratio (SNR) of the recording may be improved by filtering the data (Seymour et al., 2022). As with cryogenic MEG, OP-MEG signals of interest may be separated from surrounding noise based on their frequency and spatial topography. Model-based spatial filtering using methods such as Signal Space Separation (SSS; Holmes, Bowtell, et al., 2023; Holmes, Leggett, Hill, et al., 2025; McPherson et al., 2025; Taulu et al., 2005; Taulu & Kajola, 2005; Wang et al., 2023), Homogeneous Field Correction (HFC; Tierney et al., 2021), and Adaptive Multipole Modelling (AMM; Tierney et al., 2024) have proven highly successful in minimising interference in OP-MEG data. In addition, source reconstruction techniques such as beamformers have been shown to provide excellent suppression of extra-cranial noise (Hillebrand et al., 2023; Schofield et al., 2024). The aforementioned sensor-level spatial filters have, however, the advantage of requiring minimal assumptions about the signal of interest and no knowledge of the MEG forward model. With this in mind, here we will consider the application of AMM to reduce interference from the Neuro-1 electronics and movements through a spatially varying magnetic field. We will compare the performance of AMM with HFC, as an example of a simpler spatial filter.

AMM is a spatial filtering method where the recorded magnetic fields are modelled by spheroidal harmonics. As with SSS, harmonic functions that tend to zero at infinite distance from the origin are retained as being representative of the brain, whereas functions which tend to zero at the origin model external interference. In contrast to SSS, where both internal and external models are fit to the data, in AMM, only the internal model is fit to the data. However, this internal model is orthogonalised with respect to the external, interference space. This forms an orthogonal projection and guarantees the stability of the model for any sensor array geometry, although it comes at the cost of potential loss of brain signal for suboptimal geometries (Tierney et al., 2024). By comparison, in HFC, the external space from SSS is regressed out of the OPM data, that is, there is no internal model. Generally, in HFC, this external space is truncated to a low degree; here we will consider only the first order (homogeneous field) components. This is inherently stable and has proven highly successful in previous OPM studies (Hill et al., 2022; Rhodes et al., 2023; Seymour et al., 2021).

We also make use of the temporal extension to AMM, as described by Tierney et al. (2024). Herein, it is assumed that components of the OPM signal after interference suppression with AMM which correlate temporally with components of the original OPM data which cannot be explained by the magnetic field model underlying AMM (which describes the spatial patterns of magnetic fields from inside and outside of the head) (i.e., the model residuals) are likely caused by an artefactual source. Components of the OPM signal after AMM which correlate with the model residuals above a certain threshold (here chosen to be 0.95) are, therefore, removed from the retained signal. This is implemented through a canonical correlations analysis. The process is analogous to the temporal extension to SSS, known as tSSS. In tSSS, this approach has been shown to be highly successful for removing noise sources close to MEG sensors such as pacemakers or stimulators, which physically fall within the so-called intermediate volume, in between the volumes defined as being internal and external sources of magnetic fields, and so leak into the observed signal, despite being located outside of the brain (Taulu & Hari, 2009).

The success of spatial filtering techniques such as AMM depends on the OPM calibration (Tierney et al., 2024) and that the sensors remain within their operating range. To improve sensor calibration and increase this range, many OPMs now include closed-loop operation, where the ambient magnetic field is fed back to coils on board the sensor to minimise the field changes during the experiment (Alem et al., 2023; Labyt et al., 2019; Lee et al., 2014; Mellor et al., 2023; Sheng et al., 2017; Yan et al., 2022). Here we predominantly focus on recordings with the sensors in closed-loop operation, although open-loop data were also recorded and are shown in the Supplementary Material. Open-loop recording was feasible despite the movement interference, as the open-loop range on the Neuro-1 system at UCL is ±8 nT on one sensor axis and approximately ±16 nT on the other two axes.

To examine the movement tolerance of this OP-MEG system, we asked participants to perform an auditory oddball paradigm, which we have used previously to benchmark OP-MEG performance (Garrido et al., 2008; Mellor et al., 2023; Seymour et al., 2021) while seated and while walking around the room. The paradigm includes novel events in a series of repeated events, which violate the participant’s expectation of the tone frequency, allowing an evaluation of the mismatch negativity (MMN) response component, in addition to the auditory evoked response components from lower level sensory processing, such as the M100 component. The MMN component is observed by subtracting the response from the repeated (also called standard) tones from the novel (or deviant) tones and peaks at approximately 100–250 ms after the stimulus (Garrido et al., 2009). It is well established in MEG and in EEG (Näätänen et al., 2007, 2011, 2014; Recasens & Uhlhaas, 2017), and has been recently used to evaluate other OP-MEG systems (Demko et al., 2024; Gutteling et al., 2025).

In summary, using an auditory evoked response paradigm, we demonstrate that, with the combined use of a low-profile OP-MEG system and AMM, large translational movements of over 2 m and rotations of over 180 degrees can be well tolerated in OP-MEG. We examine the interference reduction provided by AMM for low- and high-movement conditions and discuss the impact of the closed-loop sensor operation used here.

## 2 Methods

### 2.1 Participants

Three right-handed, healthy adult participants (participant 1: 29 years, female; participant 2: 57 years, male, participant 3: 30 years, male) took part in the study. They provided written informed consent and the study was approved by the University College London Research Ethics Committee.

### 2.2 Paradigm

Participants undertook an auditory experiment while sitting stationary and while walking around the MSR at UCL (internal dimensions 3 x 4 x 2.2 m). They undertook the same experiment both while the sensors were operated under open-loop (minimum range on any sensor axis: ±8 nT, linear range: approximately ±1.5 nT) and in closed-loop (minimum range on any sensor axis: ±16 nT, linear range: approximately ±8 nT) operation. Participant 2 undertook two walking recordings with varying distances travelled, subsequently labelled recordings 2.1 and 2.2. The movement trajectory of each dataset is shown in Figure 2. Participant 3 and participant 2 for recording 2.2 were instructed to walk until they could touch the walls of the MSR. In all walking recordings, the participants rotated by at least 180°. Each run of the experiment lasted approximately 5 minutes and each participant undertook the auditory experiment at least four times (seated open loop, seated closed loop, walking open loop, walking closed loop) in a pseudo-random order (i.e., total recording time for each participant was approximately 20 minutes). The chronological recording order is given in the Supplementary Material.

The auditory paradigm followed an oddball design which we have used previously (Garrido et al., 2008; Mellor et al., 2023; Seymour et al., 2021). As before, a set of consecutive, identical tones of a particular frequency (e.g., 650 Hz) was presented to the participant, followed by a set of a different frequency (e.g., 500 Hz), and so on, for a total of 80 different sets. The tone at the start of a set is referred to as a deviant tone, while those which follow at the same frequency as the deviant are referred to as standard tones. Frequencies at 50 Hz intervals between 500 and 800 Hz inclusive were used, and selected with equal probability. All tones were 70 ms long with 5 ms rise and fall times and the number of tones in each set varied between 1 and 11. The number of tones in a set was chosen from a weighted distribution, with a 2.5% probability of 1 or 2 tones, 3.75% chance of 3 or 4 tones, and 12.5% chance of 5–11 tones. The inter-stimulus interval was 500 ms. No inter-stimulus jitter was included so that participants formed a prediction about the following tone, which would then either be confirmed if the tone was a standard, or violated if it were a deviant tone. This allowed the evaluation of the mismatch negativity (MMN) response component, considered a marker of prediction error (Huang et al., 2024). Tones were delivered via MEG-compatible air tube earphones (ER-30, Etymotic; Lucid Hearing Holding Company, LLC; TX, USA) connected to an audio card outside of the MSR. The volume of the stimuli was adjusted to be comfortable for the participant and the stimuli were presented via PsychoPy version 3.2.4 (Peirce, 2009). For each condition (e.g., walking, open loop), 80 sets of tones were included which equated to approximately 570 individual tones. For analysis of the auditory evoked response, the first 500 tones of each recording were considered and treated identically, regardless of whether the tone was at the start of a set of a given frequency (a deviant) or later in the set (a standard), in order to maximise the number of trials considered.

### 2.3 Data acquisition

#### 2.3.1 OPM system

The data were recorded with a Neuro-1 system (QuSpin; CO, USA). OPM data were sampled at 1.5 kHz, with the exception of the data from participant 3 where a temporary software change meant that they were recorded at 375 Hz. Unlike previous QuSpin electronics where the output is read as a voltage, the Neuro-1 output is digital. For open-loop operation, 24-bit digitisation was used, with the lowest bit corresponding to approximately 4 fT. This gives a noise density resolution of approximately 0.2 fT/sqrt(Hz). In closed loop, the same gain setting was used but the output was the sum of the fed-back magnetic field and OPM signal. Data were streamed over ethernet to the data acquisition computer, where they were recorded using the manufacturer’s software. To mark the beginning of the auditory tone, a 5 V pulse was sent via parallel port from the stimulus PC to the Neuro-1 data acquisition system.

In total, 56 sensors were used for each participant. All sensors were triaxial (i.e., recorded 3 channels with different orientations, labelled axes (X, Y, Z)), resulting in 168 channels. Due to abnormally high noise floors, 33, 25, and 9 channels were later rejected post-hoc for participants 1–3, respectively. The high variation in rejected channels between participants is likely due to changes in the sensor cabling and electronics between recordings. Data were recorded first from participants 1 and 2, after which the sensor cables and electronics were updated, before the recording with participant 3. This also had minor implications for the sensor positioning, which are detailed in the Supplementary Material. For all participants, the sensors were placed into participant-specific scanner casts, designed from the participants’ MRIs (Meyer et al., 2017) and coverage was across the whole head (see Fig. 1).

**Fig. 1.**
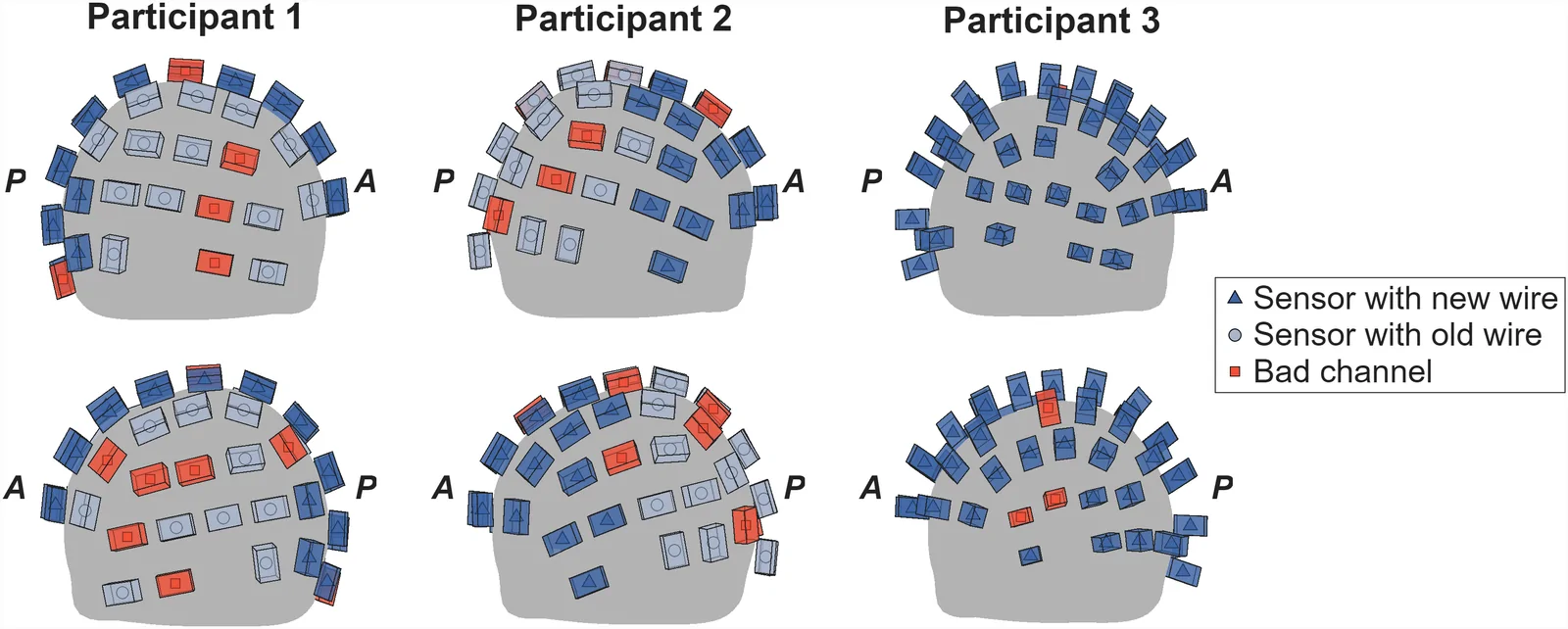
Sensor positions for each participant, showing the positions where at least one sensor channel was rejected as excessively noisy (bad channels, red squares). The wires on sensors marked with grey circles were updated between the recordings with participants 1 and 2 and the recording with participant 3.

Before the start of the experiment, the MSR was degaussed to minimise the internal magnetic field (Altarev et al., 2015). Before each experimental run, the currents on the on-board sensor coils were optimised to minimise the magnetic field at each sensor. The sensors were then calibrated using the manufacturer’s procedure and the appropriate operating mode (open or closed loop) was selected.

#### 2.3.2 Motion tracking

To quantify the degree of movement for each participant, OptiTrack Flex-13 cameras (NaturalPoint Inc.) sampling at 120 Hz were used to track the participant’s head position. The cameras work by emitting infrared light and detecting the light reflected back from retro-reflective markers placed on the scanner casts. Five, six, and four retro-reflective markers were used for participants 1–3, respectively. Six cameras (five for participant 2) were placed in three corners of the MSR, with two in each corner; one close to the ceiling and the other at mid height, with the positions optimised to maximise the field of view within the MSR. The cameras were calibrated according to the manufacturer’s procedure.

Motion tracking was synchronised with the OPM recordings by initiating the OptiTrack recording via a network connection between the cameras and a computer, immediately after which a 5 V analogue signal was sent from the computer using a National Instruments USB-6009 DAC to the Neuro-1 acquisition electronics to mark the start of the recording. This 5 V signal was then terminated at the end of the recording.

Due to the size of the MSR, some markers were intermittently out of view of the cameras. Three markers are required to reconstruct the head position and orientation. When fewer markers than this were visible, the missing data were interpolated as follows. Gaps smaller than 200 frames (1.67 seconds) were linearly interpolated in Motive, NaturalPoint’s acquisition and analysis software. Larger gaps were interpolated in Matlab using shape-preserving piecewise cubic interpolation, using Matlab’s fit function with the “pchip” model option. Motion tracking data were only used to demonstrate the degree of participant movement and not for cleaning the OPM data.

### 2.4 Data analysis

All data were analysed in Matlab 2024b, using SPM (Tierney et al., 2025) unless otherwise stated. The analysis script used can be found online (https://github.com/stephaniemellor/walking_opmeg_erf).

The OPM and motion tracking recording were aligned around the 5 V motion tracking trigger and the motion tracking data were up-sampled to the sampling frequency of the OPM data via linear interpolation (note that the OPM data were not resampled and so remain at 1500 Hz or 375 Hz, depending on the dataset). Three 5^th^ order Butterworth filters were then applied to the OPM data: a 40 Hz low-pass filter, a 2 Hz high-pass filter, and a 50 Hz notch filter to remove electronics noise. At this point, spatial filtering was applied. OPM data without any spatial filtering were compared with data where either HFC or AMM was applied. For HFC, the default implementation was used, with only homogeneous field components regressed from the data. For AMM, the SPM default model orders were used (internal order 9, external order of 2). AMM was tested both without the temporal extension and with the temporal extension with a correlation threshold of 0.95. This is implemented in SPM by setting the corrLim parameter to the chosen correlation threshold, otherwise, default parameters were used. Data were then epoched from 200 ms before to 500 ms after the analogue trigger indicating the tone onset. For analysis of the evoked response, the first 500 trials were included in each dataset. This ignores the oddball nature of the task, treating standard and deviant tones as equivalent. We additionally analysed the difference in the participants’ response to deviant and standard tones (the mismatch negativity) as described below. No trials were rejected on the basis of saturated sensors (i.e., the magnetic field exceeding the dynamic range of the sensors) or high noise levels. Such trials existed and manual rejection could have improved the SNR of evoked responses, but all trials were retained to demonstrate the feasibility of recording data despite high interference, primarily from movement.

A delay of approximately 10 ms has previously been identified between the magnetic field changing and it being observed with the QuSpin Neuro-1 system (Elzenheimer et al., 2026). We, therefore, accounted for this delay in all analyses and figures are shown with this delay corrected for. The timings given here correspond to the “true” time windows after correction, that is, if the window of interest was 50–150 ms, the OPM data recorded 60–160 ms after the trigger was used.

To test for deviation from zero, and thus to test for a significant auditory evoked response, mass-univariate, one-sample two-tailed t-tests were performed across the first 500 presented tones. These tests were performed at the sensor level, with a test at each time point and channel. Cluster-based permutation testing was performed in FieldTrip over the time window of interest (0–200 ms), in order to test for significance. To do this, a random selection of trials was inverted and a two-tailed independent samples t-test performed between the non-inverted and inverted trials for each channel and time point. All samples (i.e., each combination of channel and time point) with a p-value <0.05 were retained and clustered on the basis of temporal and spatial adjacency. Only clusters with samples from four or more channels were retained, to ensure that all clusters had samples from at least two OPM sensors (as each sensor has three channels). The sum of the t-values within each cluster was then calculated and the maximum sum of any cluster saved. This was repeated for 5000 permutations of inverted trials and a permutation distribution created from the maximum sum of the t-values within a cluster from each permutation (i.e., the test statistic was the maximum sum) (Maris & Oostenveld, 2007). In the observed data (i.e., without any trials inverted), a cluster was considered significant if the sum of the t-statistics within that cluster lay within the outer 5% (i.e., top or bottom 2.5%) of the permutation distribution.

The evoked response between 50 ms and 150 ms after the stimulus was localised through Minimum Norm Estimation in SPM. The Nolte single shell forward model was used (Nolte, 2003), based on the participant’s inner skull surface segmented from their MRI in SPM. The source space was the participant’s cortical mesh, extracted from the participant’s MRI with FreeSurfer (http://surfer.nmr.mgh.harvard.edu/), with 8004 sources with their orientation normal to the mesh surface. The power images were spatially smoothed in SPM with an 8 mm (full-width half-maximum) kernel. We extracted source-level time series at the primary auditory cortices by applying a spatial filter derived from the local lead fields, selecting the dipole orientation that maximised signal power. Where interference suppression techniques were applied to the data (HFC or AMM), the lead fields were premultiplied by the projector applied to the data, as is performed by default in SPM and FieldTrip and as described in Tierney et al. (2024).

In addition to the evoked response, we considered the participant’s mismatch negativity (MMN) response. We performed an unpaired two-tailed t-test between deviant and standard trials, assuming equal variance between groups. Standard trials were defined as the last trial of any set of tones of the same frequency, that is, if there were seven tones in a set then the first was a deviant and the seventh taken as a standard, while in the extreme case where there were two tones, the first was a deviant and the second taken as a standard. As such, for each recording, there were 80 deviant and 80 standard tones to compare.

## 3 Results

We recorded whole-head OP-MEG from three participants while seated and while walking around the MSR. During the recordings, the participants undertook an auditory oddball paradigm, with the tones delivered via MEG-compatible air tube earphones connected to an audio card outside of the MSR. Each recording was repeated with the OPMs under open-loop operation in one recording and under closed-loop operation in the other. We observed very little difference between the auditory evoked responses in open- and closed-loop operation after AMM and so focus here on results from the closed-loop recordings. Equivalent figures for the open-loop recordings are given in Supplementary Figures S9-S18 and Supplementary Table S1, and possible reasons for the similarity between the results are explored in the Discussion (Section 4).

The range of movement and magnetic field values during the walking recordings are given in Table 1, while the distributions are shown in Figure 3. The magnetic field values are given for the channel with the maximum peak-to-peak range during each recording, respectively. The area that each participant traversed is also given and was estimated by the Matlab boundary function. Only recorded positions and data from linear interpolation in Motive are used, that is, none of the data in Figure 3 or Table 1 were interpolated with Matlab. As shown in Figure 2, the distance moved varied between participants but all participants moved by at least 1 m in any given direction and rotated by at least 180° (yaw here referring to rotation around the vertical axis). Despite the variation in distance travelled, due to the rotation, differences in participant noise, and differences in interference on the different recording days, the distribution of observed magnetic field is not directly linked to distance. For example, comparing datasets from participants 1 and 3, the distance travelled by participant 3 is larger and yet the field changes observed from participant 1’s recording have a greater full width half maximum (FWHM) (Fig. 3A). Indeed, the field changes are reasonably comparable across datasets (see Fig. 3A and 3B). In all walking recordings, the magnetic field change exceeded ±4.3 nT.

**Fig. 2.**
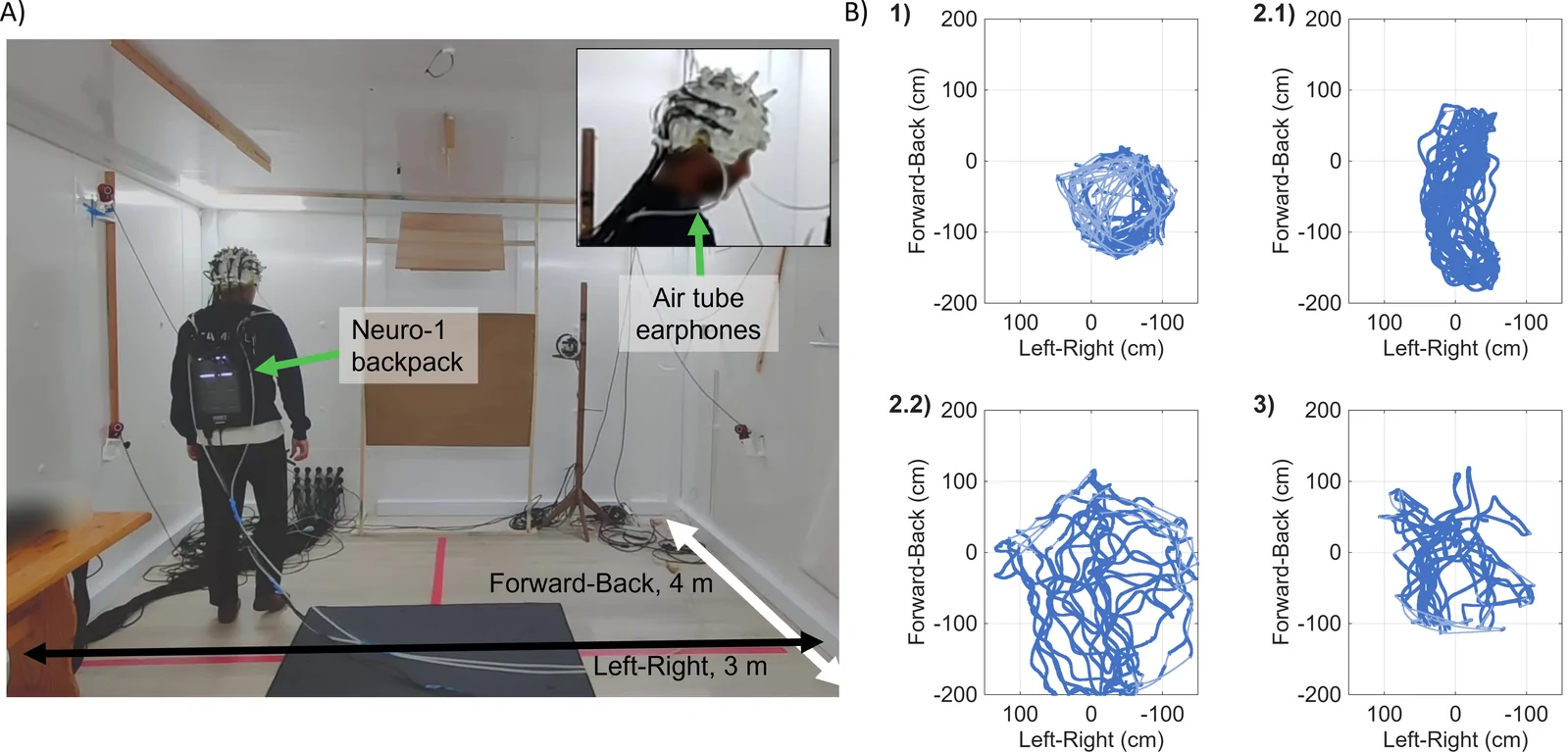
(A) Example photograph of participant walking around the MSR while wearing the Neuro-1 OP-MEG system. The insert shows a closer view of the participant wearing the OP-MEG scanner-cast, sensors, and the earphones. (B) Trajectory for each participant (1–3) for each closed-loop walking recording. The axes limits show the maximum range of movements possible within a 3 x 4 m MSR. Points indicate the centre of the cortex, estimated from the positions of the retroreflective markers placed on the head and the participants’ MRIs. Dark blue points are those where motion tracking was successful (i.e., at least 3 markers were visible to at least 2 cameras) or gaps were less than 200 frames and so were interpolated in Motive. Light blue points are those points which have been interpolated in Matlab.

**Fig. 3.**
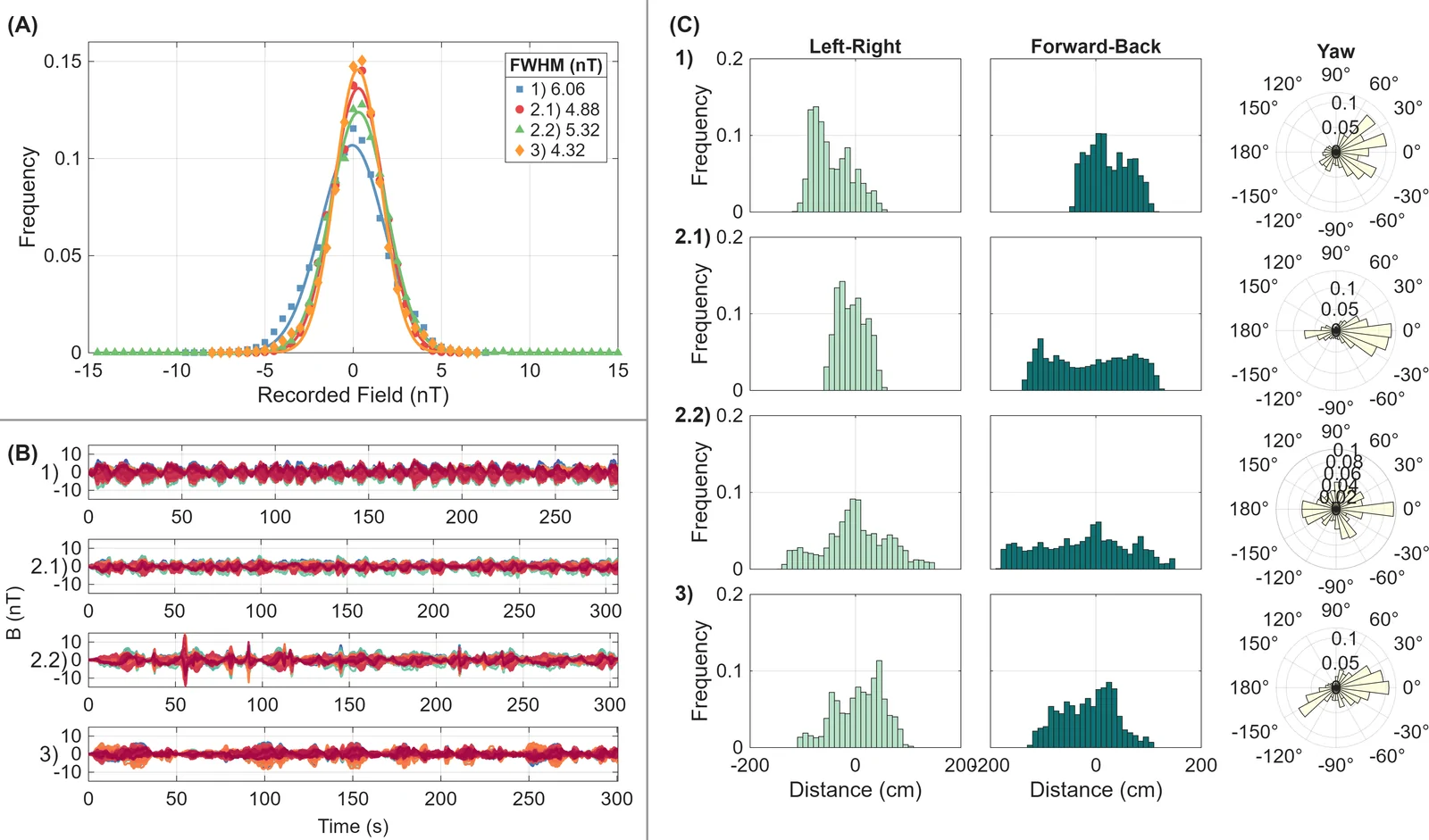
Distribution of movement and magnetic field recorded in each closed-loop walking recording. (A) Histogram (the height of each bin is given by the individual markers) and fitted normal distribution for magnetic field recorded across each walking recording. (B) Raw, unprocessed OPM walking recordings shown over time. (C) Histograms of participant displacement from their initial position in the left–right and forward–back directions and their rotation around the vertical axis (Yaw). Rotation here has been wrapped, that is, a 270° rotation is treated as a -90° rotation. (1–3) denotes the participant. (2.1) and (2.2) refer to participant 2’s two recordings with less and greater movement, respectively.

**Table 1.** Range of movement and magnetic field for each walking recording.

		Movement/Field range: Min – Max (Range)		
Participant	Area covered (m^2^)	Left–Right (m)	Forward–Back (m)	Field change (single channel) (nT)
1	1.73	-1.11 – 0.56 (1.68)	-0.46 – 1.11 (1.57)	-9.99 – 3.18 (13.17)
2.1	2.27	-0.58 – 0.55 (1.13)	-1.39 – 1.23 (2.62)	-4.60 – 4.34 (8.94)
2.2	6.33	-1.41 – 1.46 (2.87)	-1.83 – 1.49 (3.32)	-11.08 – 14.17 (25.25)
3	2.87	-1.08 – 1.09 (2.17)	-1.26 – 1.07 (2.33)	-8.42 – 6.42 (14.84)

The equivalent figure to Figure 3 for the open loop recordings is shown in Supplementary Figure S10. The open-loop range of the X axis of the sensors is -8–8 nT and is larger on the Y and Z axes (approximately ±16 nT). As a result, we rarely observed saturation of the sensors in either closed or open loop mode. As would be expected, however, the open-loop datasets show similar levels of movement but the field changes observed are lower, due to the non-linearity of the sensors in open-loop operation. Movement speed is shown in Supplementary Figures S19 and S20.

Figure 4 shows the interference suppression of HFC and AMM with and without the temporal extension, quantified by the ratio of the power spectral density (PSD) before and after the application of the spatial filter. The PSD and interference reduction shown are averaged over channels and recordings; each metric was calculated separately for each channel of each recording, then averaged over channels before averaging over recordings. Linear interpolation was used to account for the different sampling frequencies between recordings. A higher interference reduction implies that the observed recording has been reduced in amplitude. It is clear that both HFC and AMM provide considerable shielding for both the moving and stationary recordings. AMM, however, clearly outperforms HFC, with the temporal extension of particular importance at frequencies below 20 Hz.

**Fig. 4.**
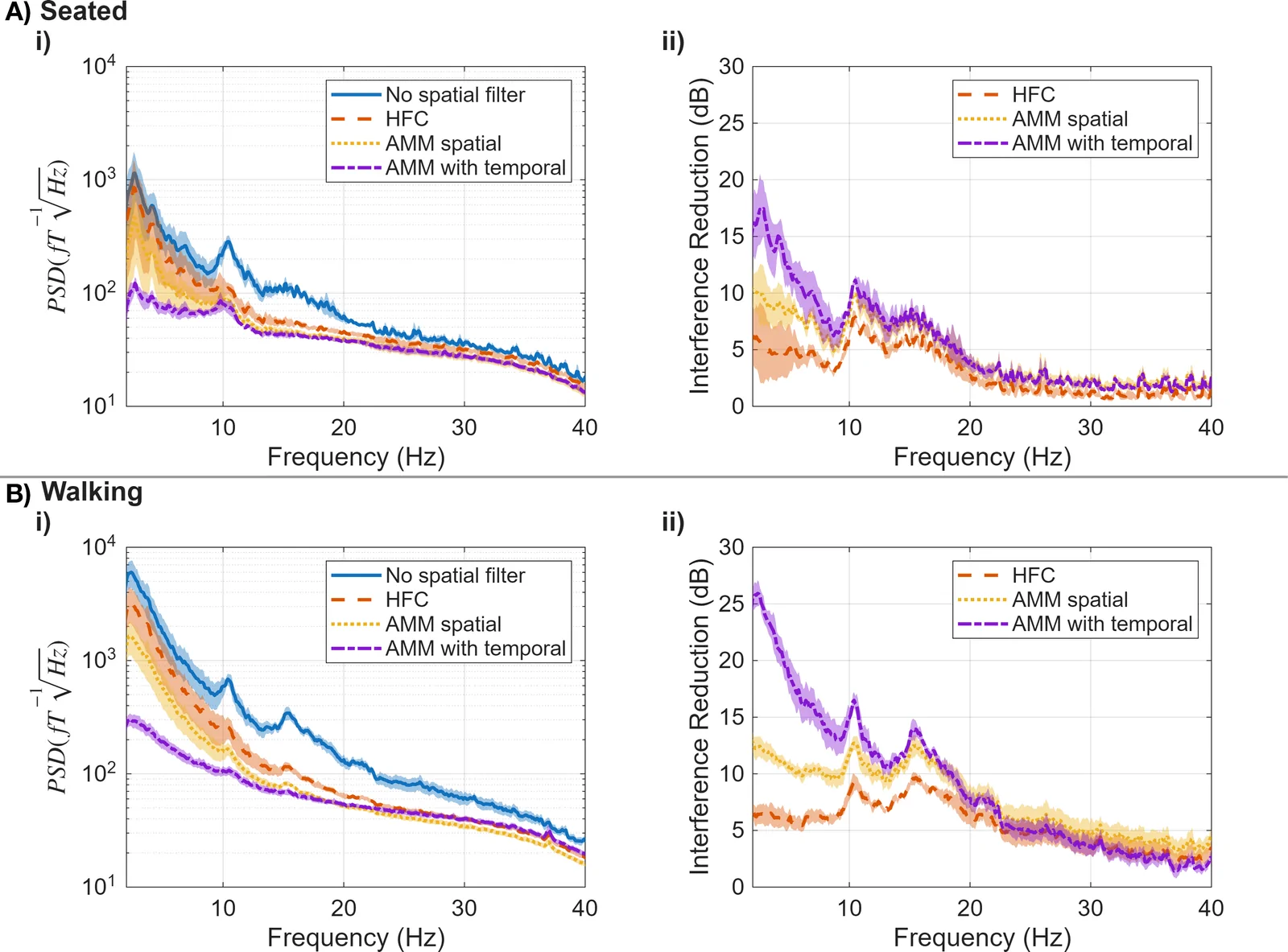
Power spectral density (PSD) (i) and interference reduction (ii) from post-acquisition application of HFC and AMM to the closed-loop data. Mean PSD curves and interference reduction were calculated over channels for each dataset separately and then averaged across datasets; the width of the curve shown here is given by the standard error of the mean across datasets. Spatial filtering algorithms reduce the PSD (relative to the data without any spatial filtering) by varying interference reduction factors, with AMM with the temporal extension leading to the highest peak interference reduction for all datasets. Data were pre-filtered to between 2 and 40 Hz and so the plots are only shown within this range. PSDs and interference reduction factors are shown separately for seated (A) and walking (B) conditions.

The benefit of this shielding from HFC and AMM is seen in Figures 5 and 6, where the auditory evoked response with differing levels of spatial filtering is presented for participant 1 (responses from other participants are given in the Supplementary Material). When participants were seated (Fig. 5; Supplementary Figs. S1 and S4), a significant auditory evoked response can be seen for all participants when AMM was included in the data preprocessing. For participant 1, the SNR (quantified by the peak t-statistic in Fig. 5A(ii)) was higher when the temporal extension to AMM was included. The amplitude of the average evoked response (Fig. 5A(i)) is similar regardless of the spatial filter applied, although the noise level visibly decreases as spatial filtering is applied, driving this increase in SNR. By comparison, in the walking condition (Fig. 6; Supplementary Figs. S2, S3 and S5), the sensor-level response cannot be observed unless AMM is applied. The peak t-statistic is lower in the walking than in the seated condition, but the signal amplitude is comparable, suggesting, as may be expected, that this decrease in t-value is driven by an increase in noise.

**Fig. 5.**
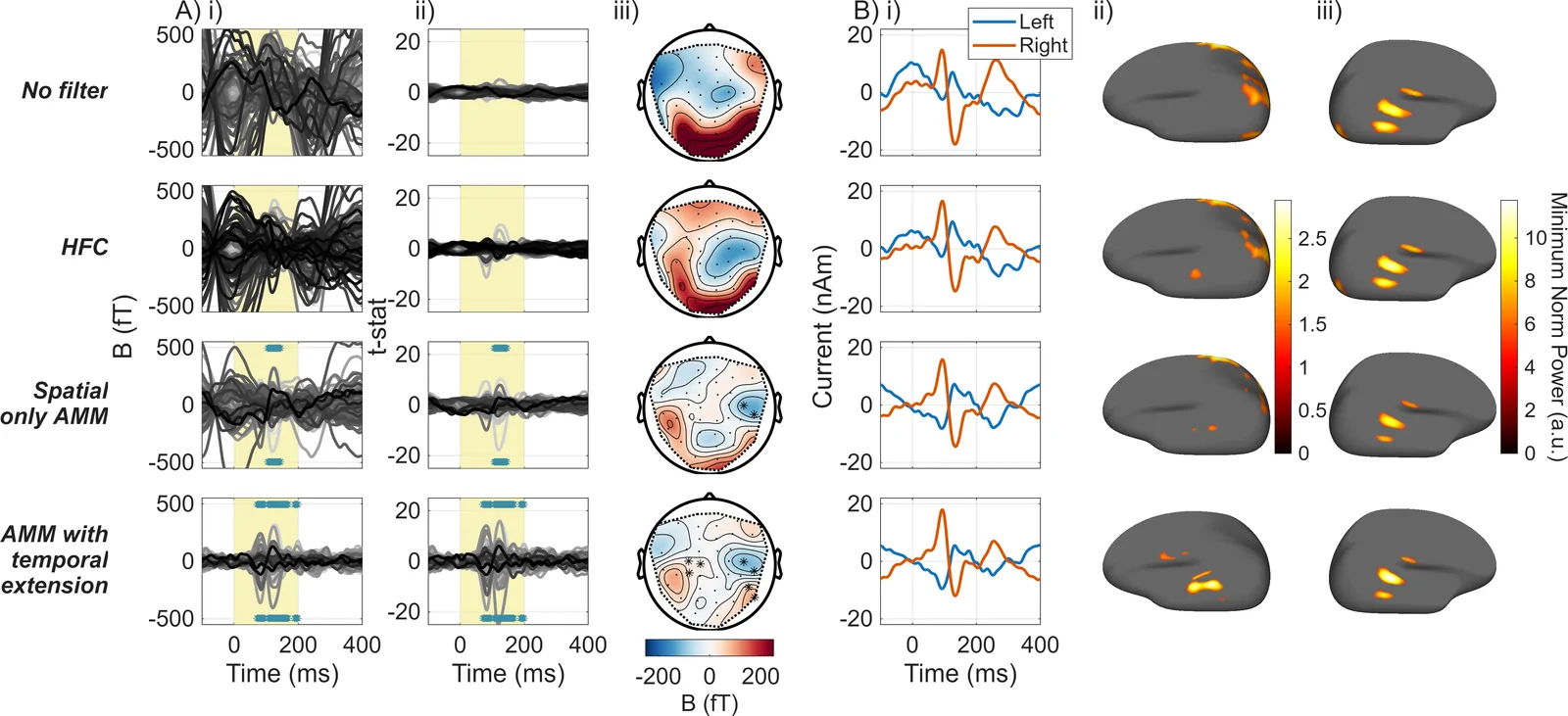
Auditory evoked responses for participant 1 while seated with the OPMs under closed-loop operation, shown after different spatial filters. (A) Sensor-level results. (i) Average evoked response in femto Tesla. (ii) Evoked response expressed as a one-sample t-statistic over trials. In both (i) and (ii), the yellow shaded area shows the time period over which permutation testing was performed. At the blue starred time points (appears as a line due to proximity of points), at least one cluster was significant at p < 0.05. (iii) The scalp topography of the average response between 80 and 120 ms. Only channels radial to the scalp are shown. Channels belonging to a significant cluster at any time during this period are shown with a star symbol. (B) Source-level results. (i) Region of interest analysis: source-level time series at the primary auditory cortices. (ii) and (iii) minimum norm localisation of the evoked response, shown in the left (ii) and right (iii) hemispheres. The maximum of the colour scale is set at the maximum power observed when AMM with the temporal extension was applied, for each hemisphere separately. The images are thresholded at 50% of this value.

**Fig. 6.**
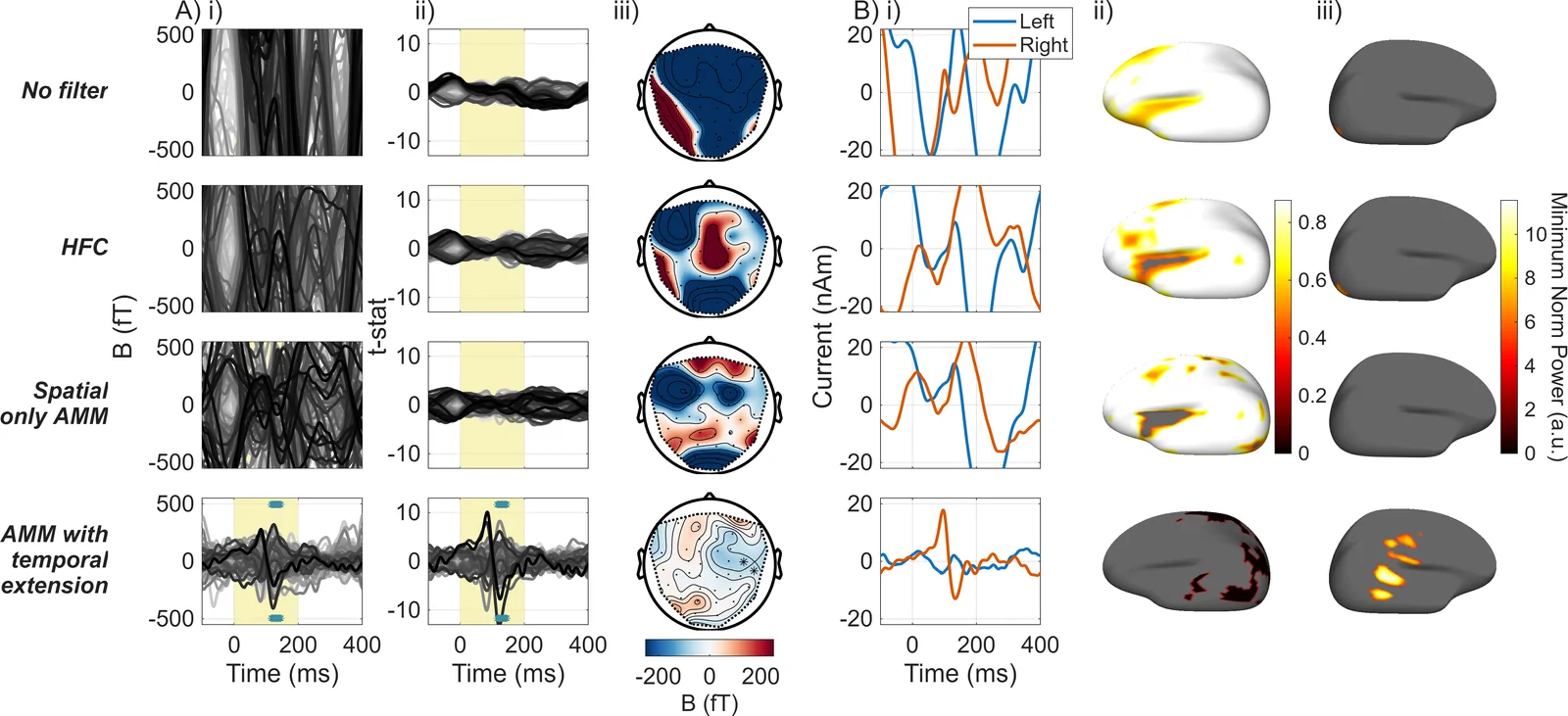
Auditory evoked responses for participant 1 while walking with the OPMs under closed-loop operation, shown after different spatial filters. (A) Sensor-level results. (i) Average evoked response in femto Tesla. (ii) Evoked response expressed as a one-sample t-statistic over trials. In both (i) and (ii), the yellow shaded area shows the time period over which permutation testing was performed. At the blue starred time points (appears as a line due to proximity of points), at least one cluster was significant at p < 0.05. (iii) The scalp topography of the average response between 80 ms and 120 ms. Only channels radial to the scalp are shown. Channels belonging to a significant cluster at any time during this period are shown with a star symbol. (B) Source-level results. (i) Region of interest analysis: source-level time series at the primary auditory cortices. (ii) and (iii) minimum norm localisation of the evoked response, shown in the left (ii) and right (iii) hemispheres. The maximum of the colour scale is set at the maximum power observed when AMM with the temporal extension was applied, for each hemisphere separately. The images are thresholded at 50% of this value.

The evoked response topographies in Figures 5A(iii) and 6A(iii) are shown at a latency of 100 ± 20 ms. The 100 ms time point was chosen a-priori due to the expected evoked response at this latency. Considering the signal at this latency rather than the latency of the peak amplitude risks missing the true peak of the activity but avoids the challenge of selecting the peak latency when the evoked response cannot be reliably observed above the level of the noise, as was generally the case in the walking condition without AMM with the temporal extension.

The source-level results are shown in Figures 5B and 6B. After AMM with the temporal extension, there is activation at or close to the superior temporal gyri both when seated and walking. There appears to be greater activity in the right than left cortex for this participant. This is also seen with the other two participants (see Supplementary Figs. S1–S5) although to a lesser degree, suggesting it may be related both to the participant and set up of the stimulus delivery. The auditory activity localises to (or close to) the right auditory cortex when seated regardless of the spatial filter. However, when walking, a response is only consistently observed at the right superior temporal gyrus when AMM with the temporal extension has been included in the data preprocessing. Similarly, a clear evoked response is only observed in the region of interest analysis when walking (Fig. 6B(i)), when AMM with the temporal extension has been used.

Figure 7 shows the MMN of all three participants when seated and walking. For brevity, only the response after AMM is included and only the OPM channel with the highest SNR evoked response 80–120 ms post-stimulus in the seated closed-loop recording is shown. The SNR was quantified by the square of the t-statistic obtained from a one-sample t-test across the first 500 tones in this recording, tested against a mean of zero. The channel with the highest absolute t-statistic at any time point in this range is presented. For participant 2, in Figure 7, the MMN is presented for the walking condition with the highest degree of movement (recording 2.2). All three participants show some evidence of an MMN response, particularly participant 2. As with the M100 evoked response component in Figure 6, the t-values are notably reduced in the walking condition, reflecting a decrease in SNR of the data. The lower number of trials here—80 by comparison with 500—makes it a more challenging response to observe in the walking data. Nevertheless, an MMN can be clearly seen for participants 1 and 2, with some indication of a response in the expected time window (100–200 ms) for participant 3.

**Fig. 7.**
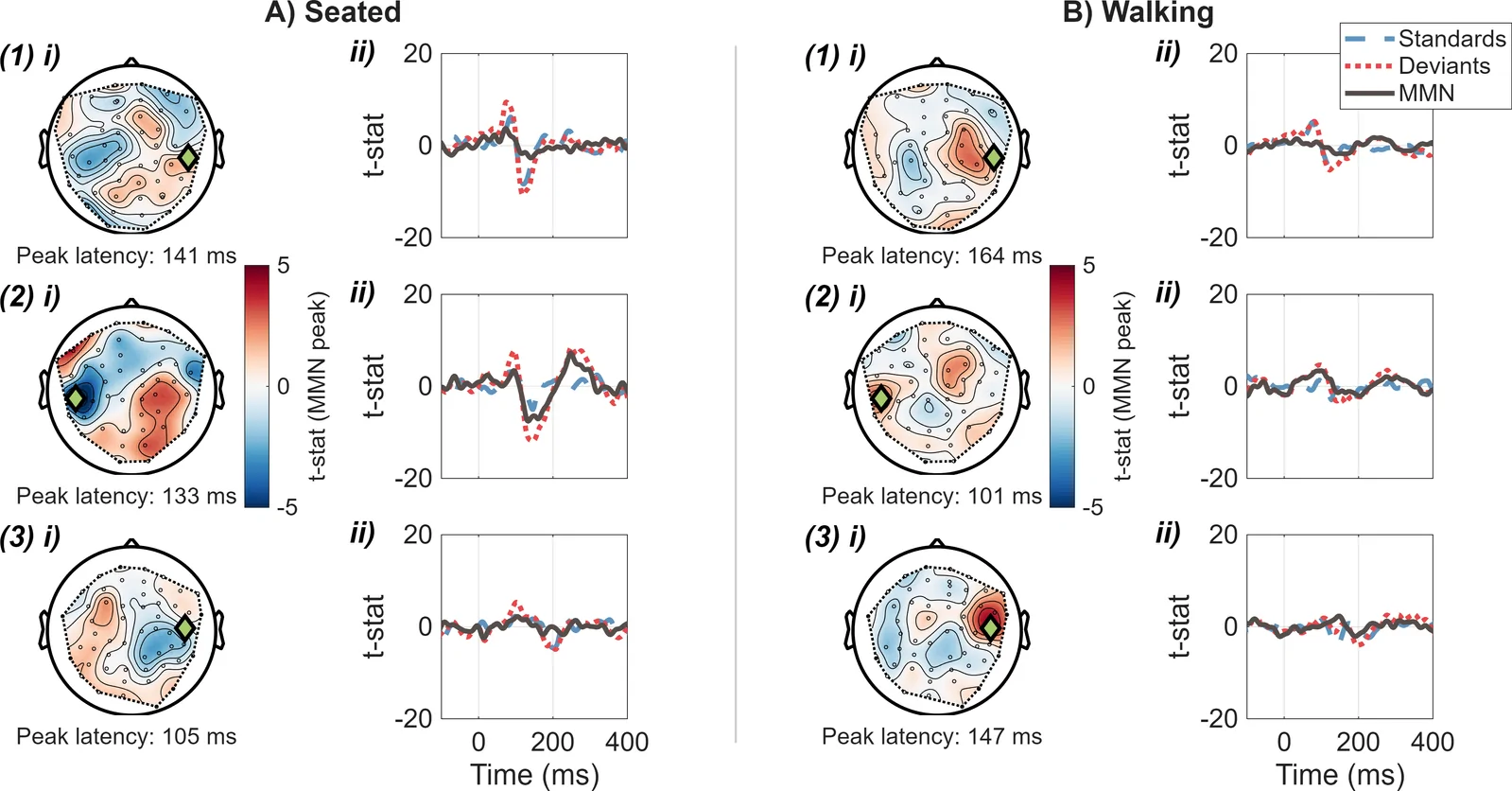
Mismatch negativity (MMN) response for all participants (1–3) while (A) seated and (B) walking, after AMM with the temporal extension. For participant (2), the walking condition covering the greater surface area is shown (i.e., recording 2.2). The topography (i) is shown at the latency of the peak MMN between 100 and 275 ms on the channel with the highest SNR auditory evoked response, shown with a green diamond symbol in the topography. The time series of the MMN at this channel (ii) is shown as a t-statistic.

## 4 Discussion

We have shown that sensor-level evoked responses can be recovered from OP-MEG data recorded while participants walked around an MSR, traversing at least 1.7 m^2^ and rotating by over 180°, incurring field changes of up to 25.25 nT (maximum single channel peak-to-peak range). We found that AMM is effective at separating movement-related interference from the brain signal of interest and consistently outperformed HFC based on the sensor-level t-statistics in an auditory evoked response paradigm.

The spatial filters tested here are all simple to use interference suppression methods for OP-MEG. Both HFC and AMM are based on harmonic models of the MEG signal and interference. These models are based on the physics of magnetic fields and so are interpretable and performance does not rely on any previous OPM data or knowledge of the underlying anatomy. In other words, such methods are attractive as they are relatively assumption free with guaranteed stability for any OPM array, conditional on a sufficient number of channels to model the internal space in the case of AMM (≥99 with the default parameters). This is important in situations in which there is no spatial hypothesis and is particularly useful for analyses of events well described at sensor level (e.g., epileptogenic spike localisation). Similar approaches are well established within cryogenic MEG; signal space separation (SSS) is regularly used in MEGIN systems to minimise background interference. Here, we demonstrate that the application of these relatively simple techniques is sufficient to minimise the interference observed in ambulatory OP-MEG recordings to allow reliable auditory evoked responses to be recovered, despite the high degree of movement.

During the experiment, participants were asked to walk naturally around the room, rather than along a particular path. This was done to reflect the kinds of movements that participants might be expected to make during naturalistic neuroimaging experiments, one of the key potential applications of OP-MEG. Interestingly, although all datasets have different walking trajectories, the change in magnetic interference observed did not directly relate to the distance travelled. This could be due to a number of factors, including the inhomogeneous shielding factors across the space due to the passive shielding, field changes due to mechanical vibration of the room due to participant heel strike, relative movement of the OPM sensors and backpack during walking, and similar levels of head rotation during the recordings. Additionally, as the data were acquired from each participant on different days, as well as each participant being a different height, the environmental interference will have varied between participants.

In the results presented, participant 3 shows the smallest responses of the participants. We believe that this is simply due to individual differences but there were some updates to the system between participants 2 and 3 which could affect the SNR of the observed response. The main difference is the orientation of the OPMs (which were rotated by 90°, as shown in Figure 1, and so a different OPM axis was radial to the scalp). Additionally, for participants 1 and 2, approximately half of the sensors were connected to the Neuro-1 backpack through flat ribbon cables, while the other half, which were newer, were connected with round cables. For participant 3, the ribbon cables were updated to round cables. The acquisition software was also updated, which led to a change in the sampling rate. It is common in other setups with the same OPM system to record with the OPMs in this orientation (e.g., Sanders et al., 2025; Schofield et al., 2024); the wires were only updated in line with the other half of the sensors, which had been used successfully for participants 1 and 2; and the changes to the acquisition programme were predominantly related to the user interface rather than the way in which data were acquired. Therefore, we believe that the differences in the observed signals between participants were driven by individual differences, rather than the recording setups.

We note that, particularly in the walking condition, the temporal extension of AMM was key to the observed improvement in the SNR of the sensor-level evoked responses by comparison with the raw data. Both HFC and AMM were beneficial for the observed evoked responses in both the seated and walking conditions, but AMM with the temporal extension was, for these data, necessary for observable responses for all walking datasets. The improvement of purely spatial AMM over HFC is most likely due to the higher complexity of the model: in HFC, only homogeneous interference was modelled and regressed from the recordings, while in AMM, the signal of interest, homogeneous interference, and interference with a spatial gradient were modelled. HFC can be extended to additionally model interference with a spatial linear gradient, the effect of which is tested here in Supplementary Figures S6–S8. When extended to include linear gradient terms, the performance of HFC is far more comparable with that of (spatial only) AMM. The further improvement of AMM when using the temporal extension suggests that there are interference components which cannot be explained with these spatial models. These components could be due to higher order external interference or imperfections in the measurement system due to imperfect geometry (Hill et al., 2025) or non-linearities within the sensor array (Borna et al., 2022; Iivanainen et al., 2019).

While practically we found AMM with the temporal extension to be of great benefit for interference suppression in these data, it does have some theoretical limitations. Firstly, sufficient OPM channels are required in order to use a sufficiently complex model of the magnetic field from the brain to capture the expected spatial patterns (as there should always be more observations than model parameters). As shown by Tierney et al. (2024), a 9^th^ order internal model is generally sufficient (as was used here and as is the default implementation in SPM 25 (Tierney et al., 2025)): if a simpler model is used, the signal will effectively be spatially smoothed. Such a 9^th^ order model has 99 components, and, therefore, requires a minimum of 99 channels, with ideally more for optimal performance. Secondly, as shown in Tierney et al. (2024), the quality of AMM external noise rejection is a reciprocal of nonlinearity in the recording system: as nonlinearity increases, the interference suppression provided by AMM decreases. These errors could stem from inaccuracies in the sensor gain, positions or orientations, which have been previously shown to be inaccurate for OP-MEG without appropriate calibration (Borna et al., 2022; Hill et al., 2025). The temporal extension, however, does not rely on assumptions of the sensor positions and so is expected to reduce the impact of such nonlinearities where they exist. The main limitation of this temporal extension is that the correlation limit must, at least currently, be chosen manually, and so is dependent on the user. If too low of a correlation limit is chosen, it is possible that signal will be removed from the recording. This choice can be made systematically based on the SNR of the data, as outlined by Tierney et al. (2024), although this does, naturally, necessitate an estimate of the SNR.

This paper is based on two previous studies from our group (Mellor et al., 2023; Seymour et al., 2021). The key differences are in the degree of movement, particularly of rotation, the ambient magnetic fields, and sensor electronics. We found that placing the sensor electronics in a backpack had considerable advantages for participant movement, greatly decreasing the number of cables and reducing the consequent physical limitations. Participants were able to walk and rotate further than previously: where before rotation was limited to ±90° and movements within the front approximately 2 m of the MSR due to the cable length, participants were now able to rotate by over ±180° and use the entire space (MSR internal dimensions 3 x 4 x 2.2 m), going up to and touching the MSR walls. This led to interference field changes of up to 25.25 nT range, approximately 3 times the ±4.5 nT limit which was available for the previous QuSpin QZFM Gen-2.0 sensors used at the time. In previous studies, particularly noisy trials have been rejected and removed from the final analysis. Here, due to novel interference correction techniques, we show that we are able to retain all trials, demonstrating the viability of OP-MEG for more complex naturalistic tasks than have previously been undertaken.

Despite the clear advantages of the updates to the sensor electronics in terms of participant movement, we did observe interference in the seated recordings which we expect to be caused by the proximity of the OPM electronics. This interference was sufficient to impact the SNR of the evoked responses by comparison with previously recorded OP-MEG auditory evoked responses from the same paradigm (Mellor et al., 2023; Seymour et al., 2021) but was reduced with AMM. Without any interference suppression, in the seated, stationary case, significant evoked responses could not be observed for all participants at the sensor level here. This could naturally be minimised by moving the backpack further from the participant when not needed but is a necessary compromise if wearing the sensor electronics facilitates novel experimental paradigms. It is promising to observe the interference suppression provided by AMM for this proximal source of interference, opening up the possibility of future experiments with not just sensor electronics, but also other sources of interference including stimulation electronics (Mardell et al., 2024) or metal implants close to the sensors.

We recorded data with the OPMs under both open- and closed-loop operation. Due to the relatively large (±8 nT on X-axis and approximately ±16 nT on the Y and Z sensor axes) open-loop range of the sensors, we observed very little saturation of the sensors, unlike previous studies (Mellor et al., 2023). Nevertheless, as expected, we observed smaller interference field strengths under open-loop operation for the same movements due to the non-linearity of the sensors (Iivanainen et al., 2019; Tierney et al., 2019). It has been previously shown that the QuSpin OPMs experience smaller calibration and cross-axis projection errors (CAPE) (Borna et al., 2022) when under closed-loop operation (Schofield et al., 2024). In spite of this, we did not see a considerable difference between the closed-loop and open-loop evoked responses after the application of AMM. There are multiple potential explanations for this. One could be that the temporal component of AMM does not make any assumptions about the sensor linearity (whereas the spatial component does). It may also be that the errors in the output of AMM are dominated by geometric errors in the sensor positions and orientations, and so sensor gain errors are comparatively unimportant. This can be minimised going forward with novel sensor localisation methods (Hill et al., 2025) and as such the use of closed-loop sensors may have a greater impact on observed responses in future OP-MEG systems.

In conclusion, we have shown that auditory evoked responses can be recorded with OP-MEG during large participant movements over 1.7 m^2^, with 180-degree rotations, leading to field changes of up to 25.25 nT peak-to-peak range. This study demonstrates the importance of interference suppression using algorithms such as AMM for such recordings and shows the feasibility of recording with the OPM electronics held on the participants’ back. This shows the suitability of OP-MEG for research involving ambulatory movement, including naturalistic paradigms. Such a naturalistic approach provides a more ecologically valid platform for neuroscience research, particularly in areas such as spatial navigation and memory (Maoz et al., 2023). Additionally, a greater movement tolerance suggests the value of OP-MEG for recordings from populations who may have difficulty remaining still, such as paediatric populations.

## Supplementary Material

Supplementary Material

## Data Availability

Anonymised data are available on Zenodo (https://doi.org/10.5281/zenodo.15229491). The analysis scripts used in this manuscript are available on GitHub (https://github.com/stephaniemellor/walking_opmeg_erf).
