# Supplementary material for: Auditory evoked responses during ambulatory OP-MEG
Source: Imaging Neurosci (Camb). 2026 Nov 3;4:IMAG.a.1424. doi: 10.1162/IMAG.a.1424 (PMC13732490; doi:10.1162/IMAG.a.1424)
Supplement: Supplementary file 1 [file IMAG.a.1424_supp.pdf]

# Supplementary Material for "Auditory evoked responses during ambulatory OP-MEG"

## 1 Auditory Evoked Responses from Participants 2 and 3

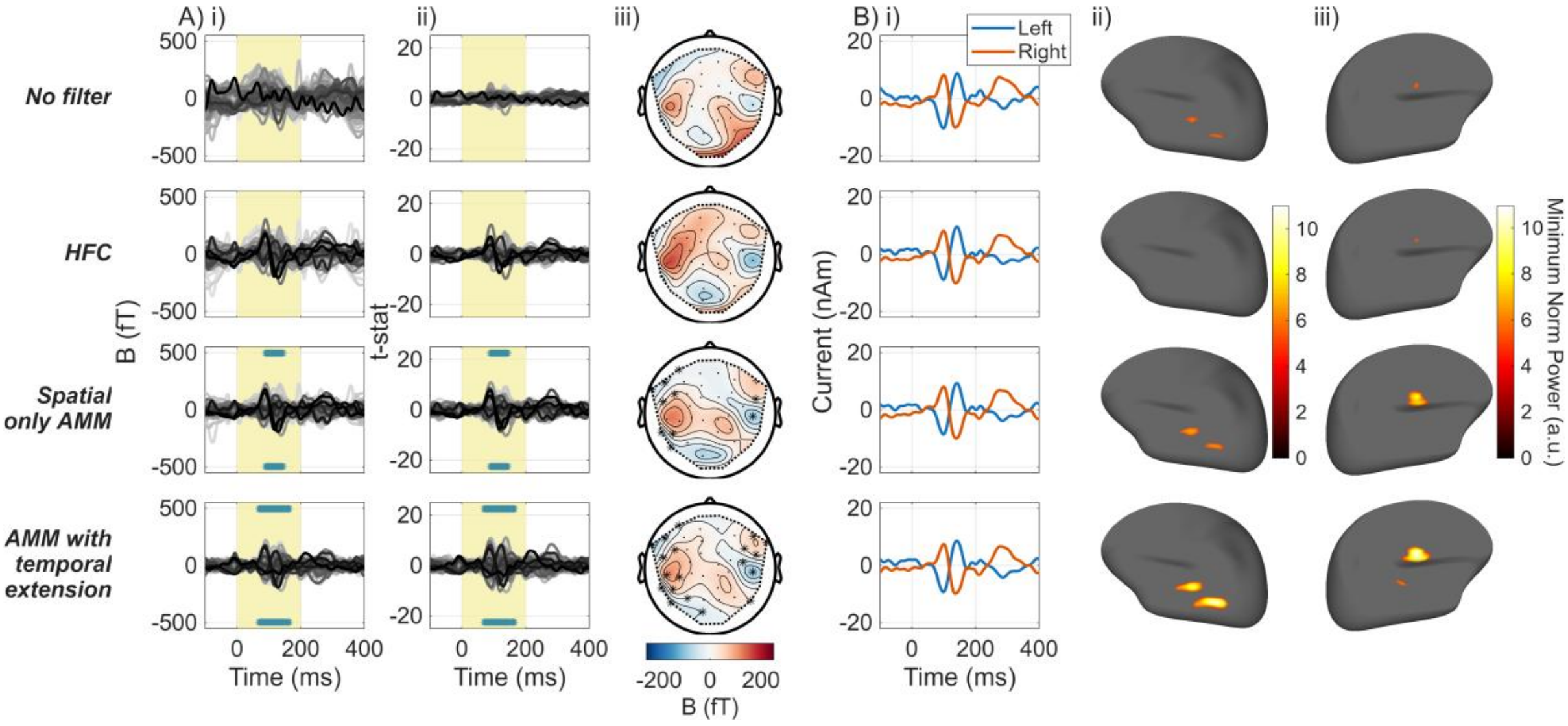


*Supplementary Figure 1. Auditory evoked responses for participant 2 while seated with the OPMs under closed-loop operation, shown after different spatial filters. See Figure 5 of the main manuscript for full caption.*

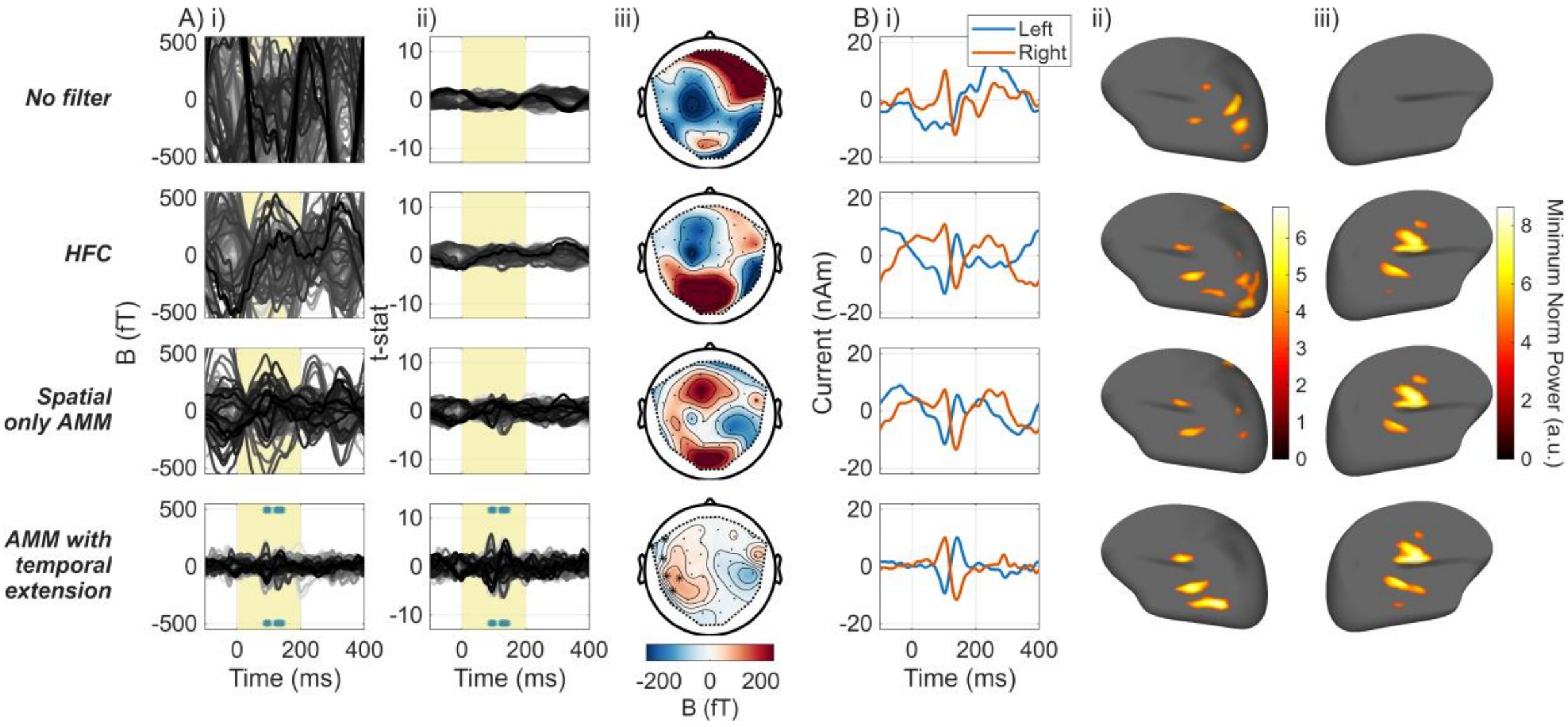


*Supplementary Figure 2. Auditory evoked responses for participant 2 while walking recording 2.1 with the OPMs under closed-loop operation, shown after different spatial filters. See Figure 5 of the main manuscript for full caption.*

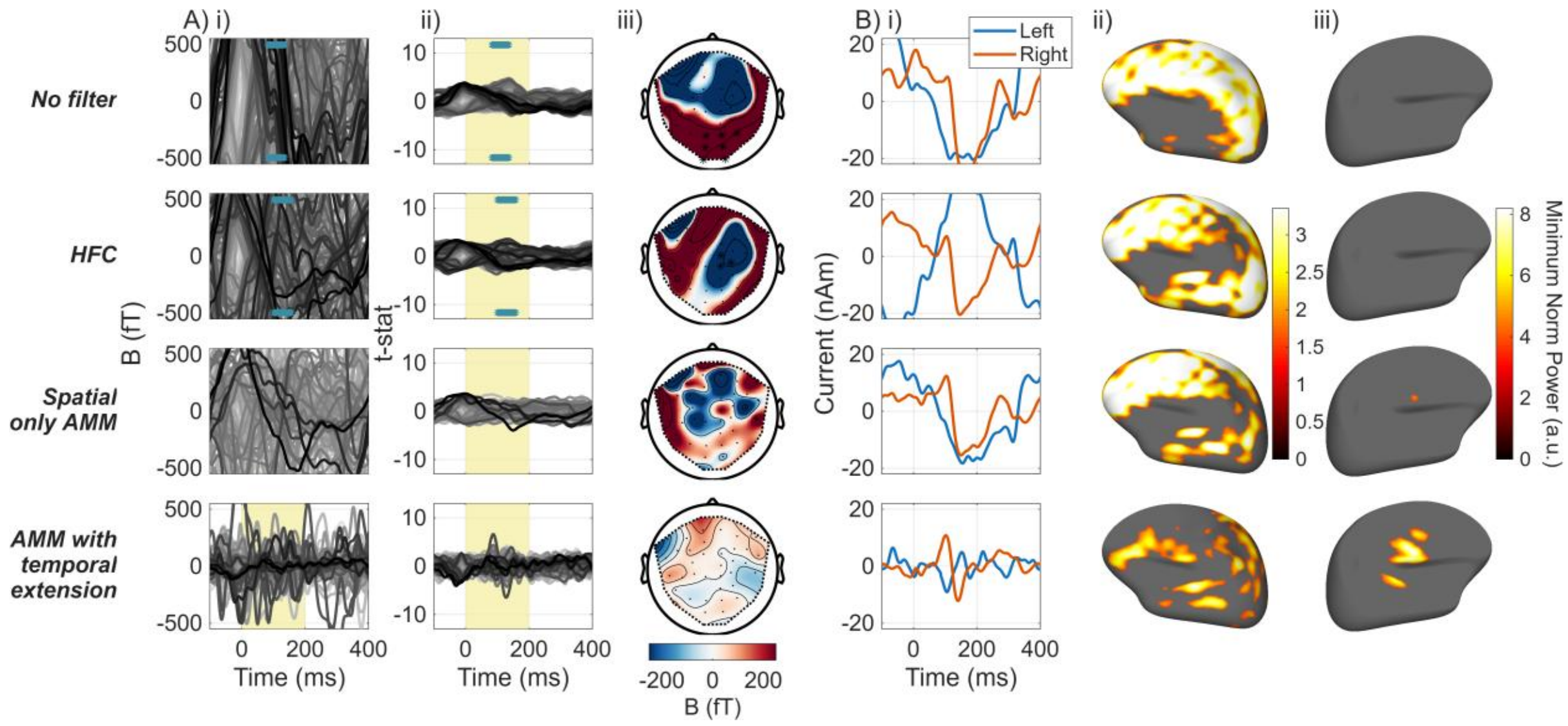


*Supplementary Figure 3. Auditory evoked responses for participant 2 while walking recording 2.2 with the OPMs under closed-loop operation, shown after different spatial filters. See Figure 5 of the main manuscript for full caption.*

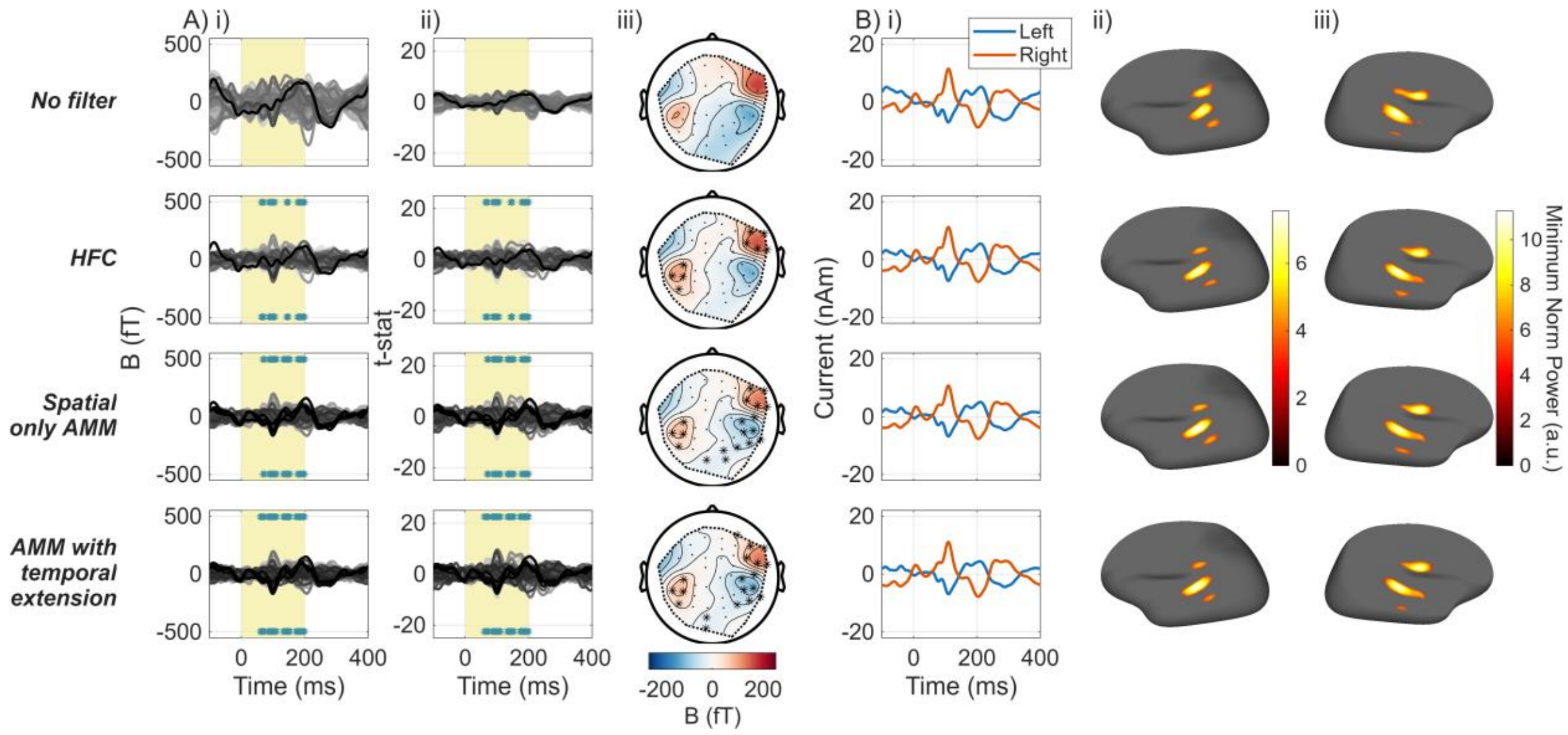


*Supplementary Figure 4. Auditory evoked responses for participant 3 while seated with the OPMs under closed-loop operation, shown after different spatial filters. See Figure 5 of the main manuscript for full caption.*

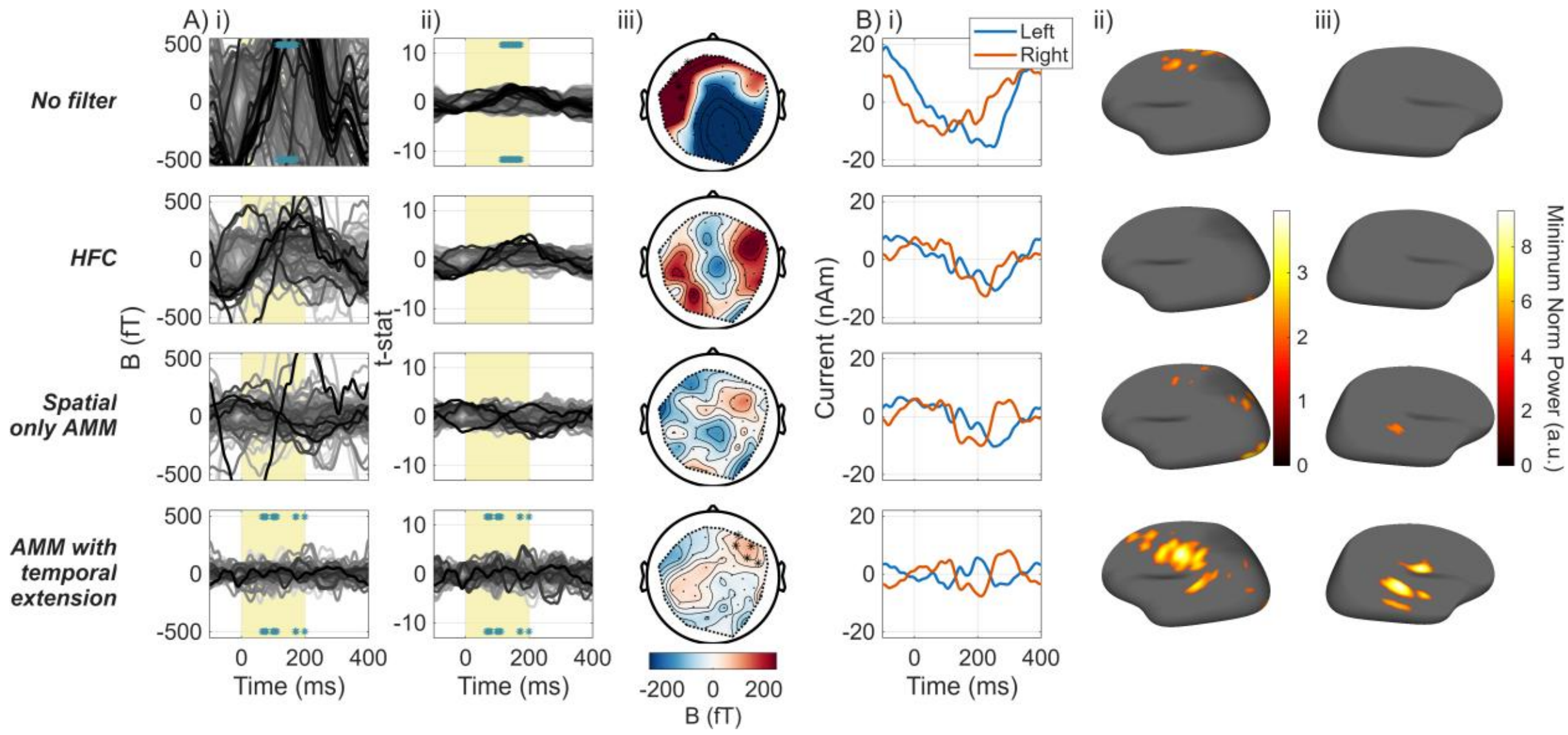


*Supplementary Figure 5. Auditory evoked responses for participant 3 while walking with the OPMs under closed-loop operation, shown after different spatial filters. See Figure 5 of the main manuscript for full caption.*

# 2 Sensor Positioning and Cabling

The same OPMs were used for each recording. However, the sensor cabling and electronics were updated between the recordings with participants 1 and 2 and participant 3, which was recorded at a later date. For participants 1 and 2, 24 of the sensors used had thinner sensor wires than previous generations of QuSpin OPMs. These sensors should experience smaller cable artefacts as a result but, due to the changed shape of the connector between the sensor and the cable, did not fit perfectly into the scanner-cast slots and so there is higher uncertainty on their orientation (~+5° around the tangent to the scalp surface) and position (~+2 mm radially from the scalp surface). These orientation errors are however marginal in comparison to the expected deviation between case and sensing geometry (Hill et al., 2024). The positions of all sensors are shown in Figure 1 of the main manuscript, where these sensors with updated cabling are marked in dark blue with a triangle symbol. For participant 3, all cable wires were these newer thin cables and an updated scanner-cast design was used which could accommodate this cabling, removing this position and orientation uncertainty.

# 3 Recording order for each participant

The chronological order of each recording (i.e. open-loop walking vs. closed-loop sitting) is given below.

Participant 1:

1. Seated closed-loop
2. Seated open-loop
3. Walking open-loop
4. Walking closed-loop

Participant 2:

1. Walking (to walls – recording 2.1) closed-loop
2. Seated open-loop
3. Seated closed-loop
4. Walking (to walls – recording 2.1) open-loop
5. Walking (centre of the room – recording 2.2) open-loop

6. Walking (centre of the room – recording 2.2) closed-loop

Participant 3:

1. Seated open-loop
2. Walking open-loop
3. Walking closed-loop
4. Seated closed-loop

# 4 HFC with gradient terms

In the main manuscript, HFC was implemented with the default parameters, meaning that only spatially homogeneous interference terms were modelled and regressed from the data. The method can be extended to model higher order (i.e. spatially more complex) external interference. Here we test the performance of HFC for interference suppression in this data when linear gradient terms are included in the model, i.e. the interference model for HFC is extended to be the same as that used for AMM in the main manuscript.

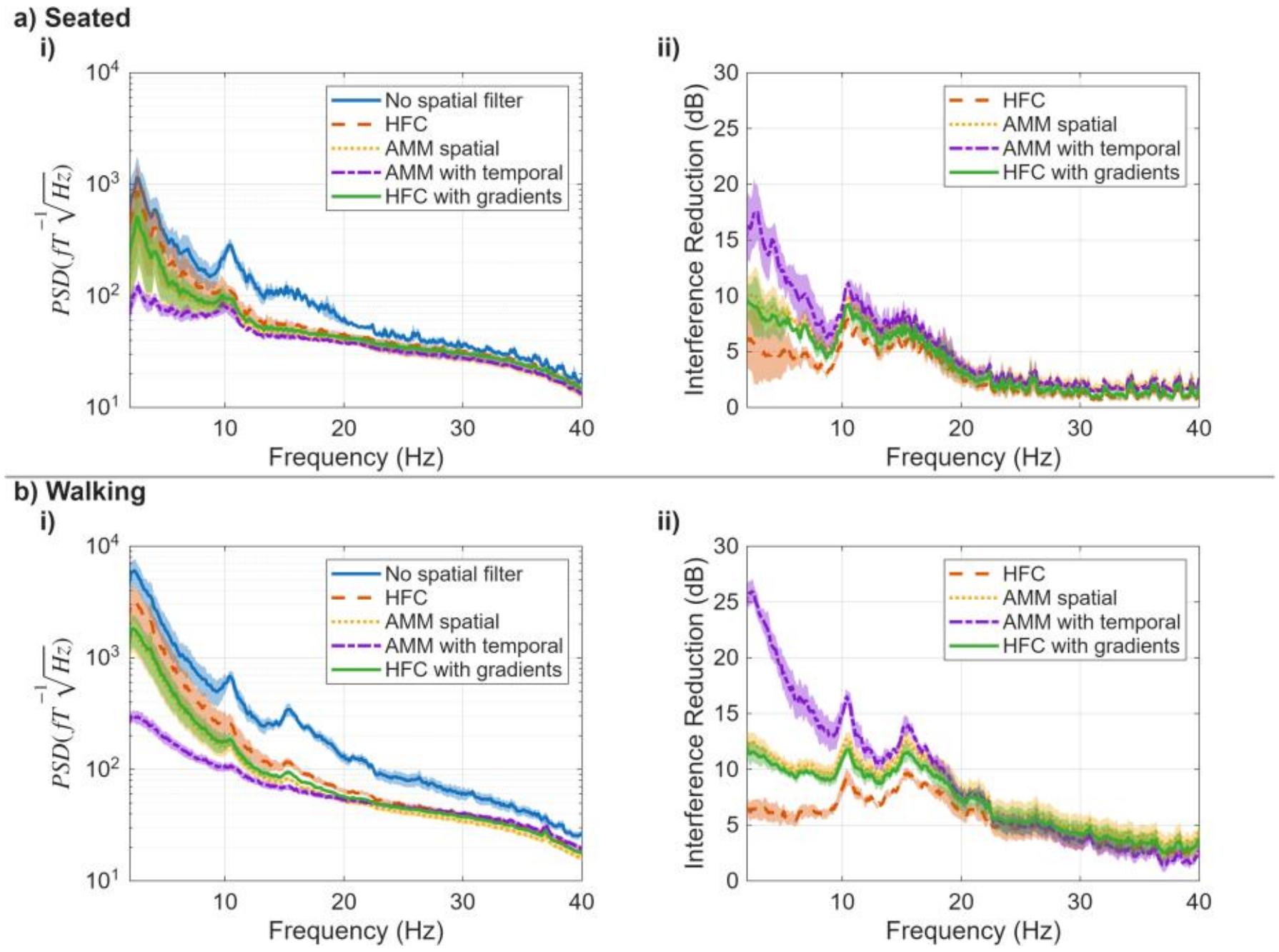


*Supplementary Figure 6. Power spectral density (PSD) (i) and interference reduction (ii) from post-hoc application of HFC and AMM to the closed-loop data. The figure is the same as Figure 4 of the main manuscript, with the addition of HFC with linear gradients as a spatial filter. Mean PSD curves and interference reduction were calculated over channels for each dataset separately and then averaged across datasets; the width of the curve shown here is given by the standard error of the mean across datasets. Spatial filtering algorithms reduce the PSD (relative to the data without any spatial filtering) by varying factors, with AMM with the temporal extension leading to the highest peak interference reduction for all datasets. Data were pre-filtered to between 2 Hz and 40 Hz and so the plots are only shown within this range. PSDs and interference reduction factors are shown separately for seated (a) and walking (b) conditions.*

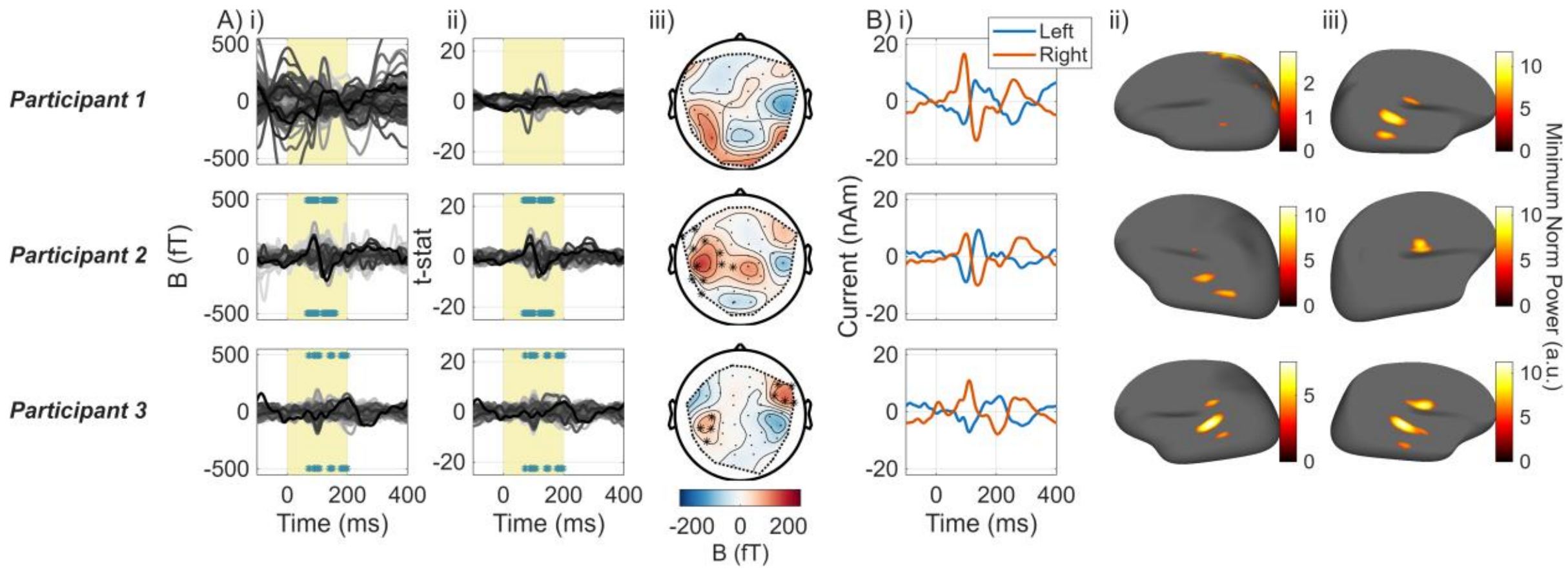


*Supplementary Figure 7. Auditory evoked responses while seated with the OPMs under closed-loop operation, after HFC with linear gradient terms. Different rows show the response from different participants. See Figure 5 of the main manuscript for full caption.*

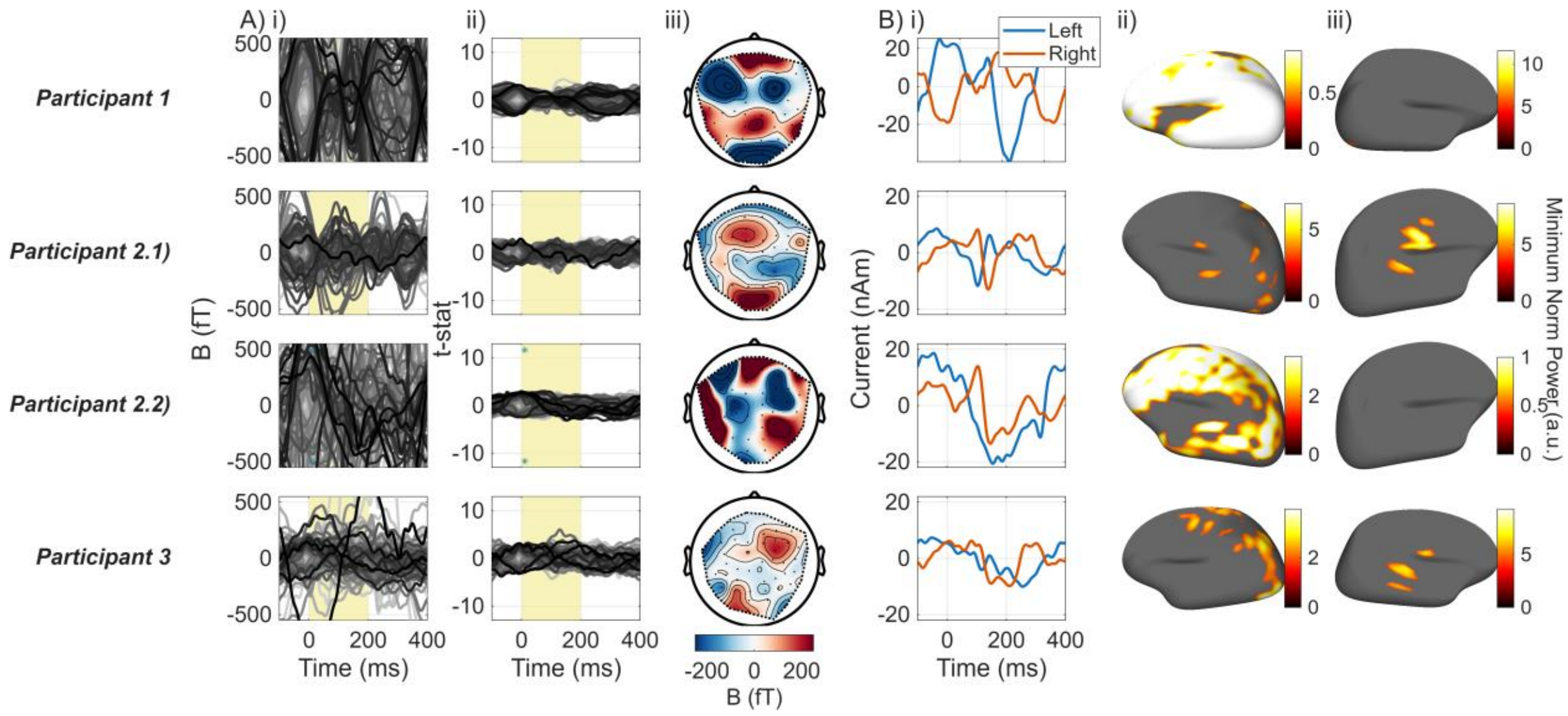


*Supplementary Figure 8. Auditory evoked responses while walking with the OPMs under closed-loop operation, after HFC with linear gradient terms. Different rows show the response from different datasets. See Figure 5 of the main manuscript for full caption.*

# 5 Open Loop Recordings

In this section, we report the findings from the open-loop recordings. Each figure is the equivalent of one for the closed-loop recordings given in the main manuscript.

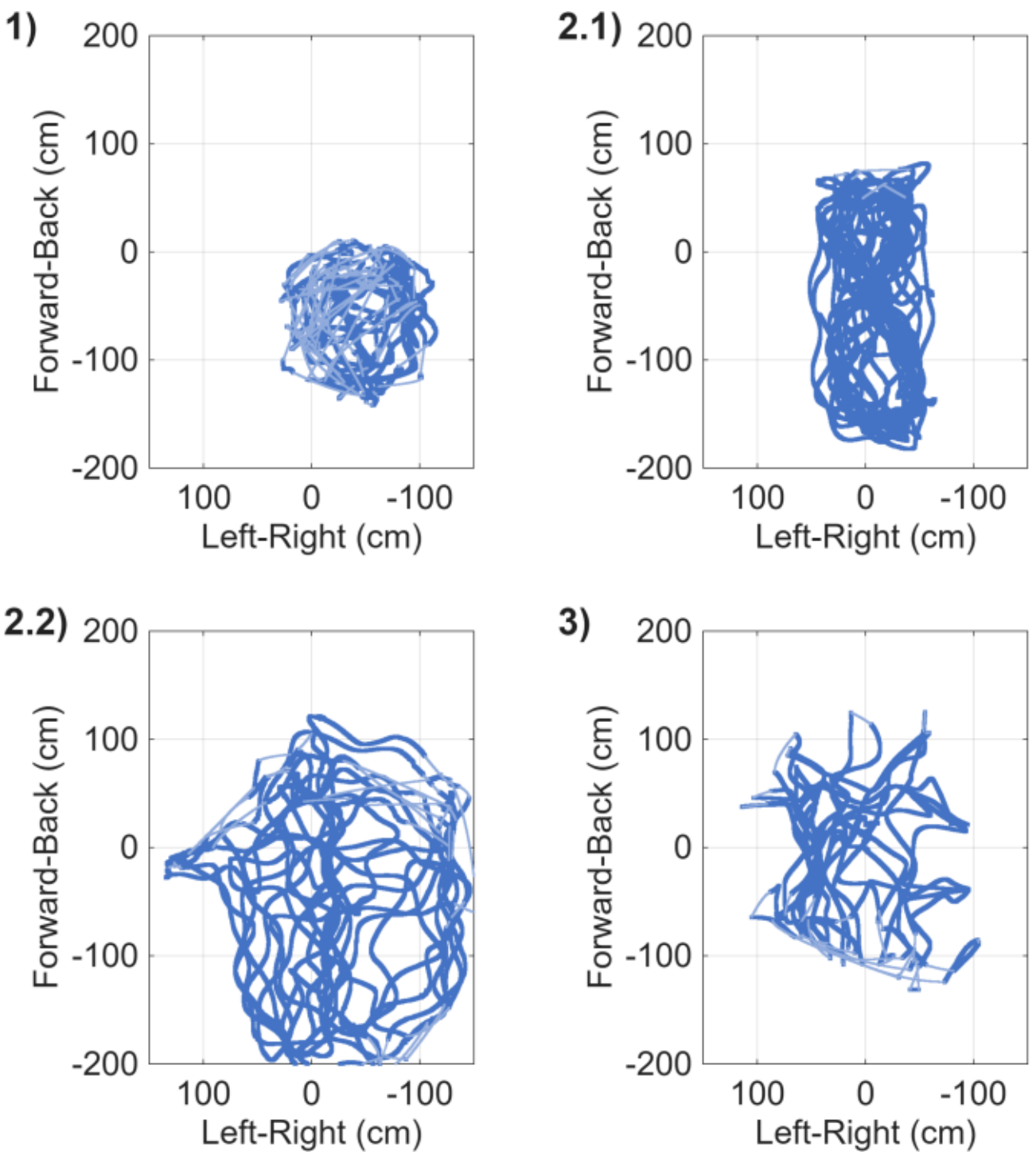


*Supplementary Figure 9. Trajectory for each dataset (A–D) for each open-loop walking recording. Points indicate the centre of the cortex, estimated from the marker positions placed on the head and the participants' MRIs. Dark blue points are those where motion tracking was successful (i.e. at least three markers were visible to at least 2 cameras), light blue points are those which have been interpolated. Closed-loop trajectories are shown in Figure 2 of the main manuscript.*

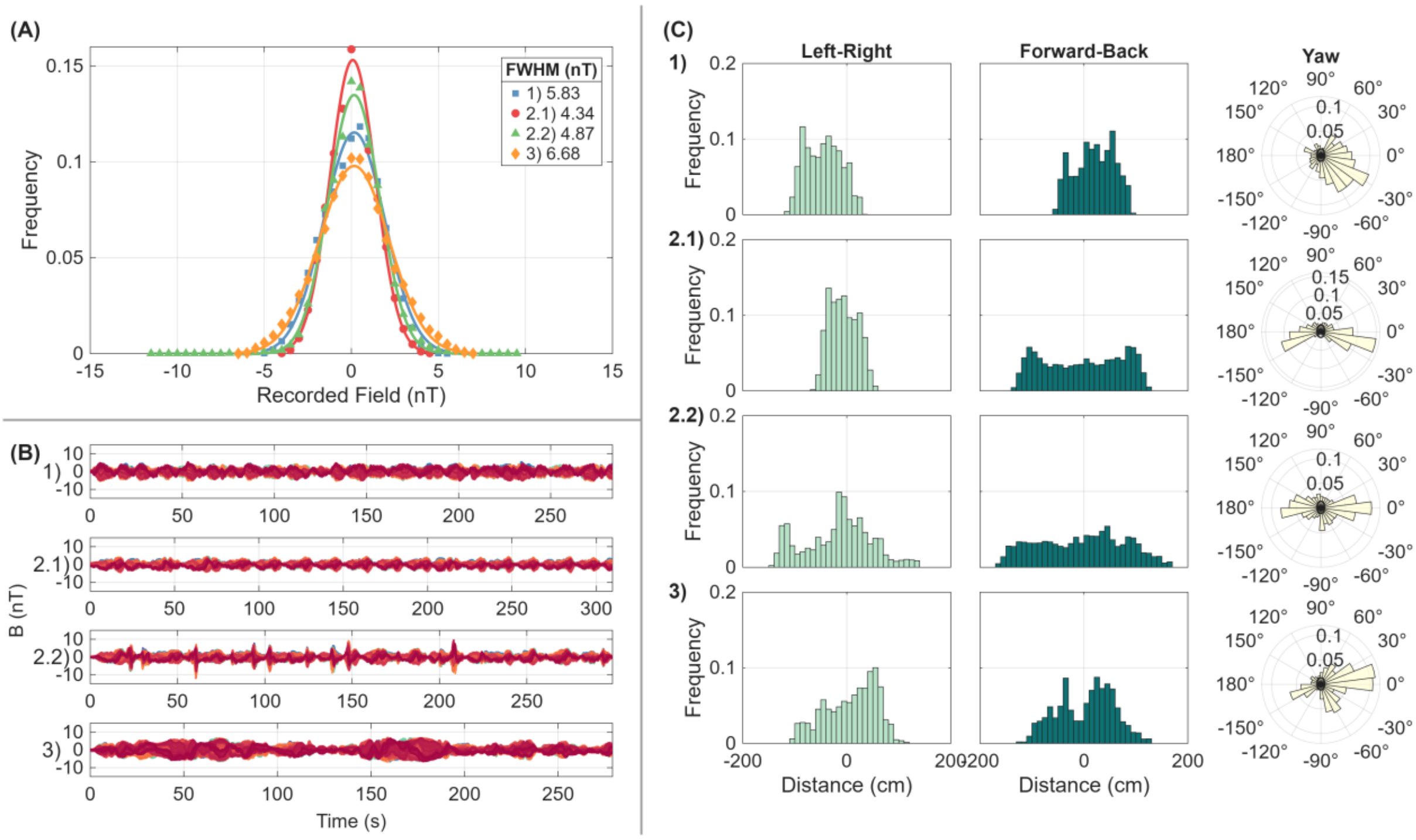


*Supplementary Figure 10. Distribution of movement and magnetic field recorded in each open-loop walking recording. (1) Histogram (individual markers) and fitted normal distribution for magnetic field recorded across each walking recording. (2) Raw, unprocessed OPM walking recordings shown over time. (3) Histograms of participant displacement from their initial position in the left-right and forward-back directions and their rotation around the vertical axis (Yaw). A–D denotes the dataset. Equivalent for closed-loop recordings is shown in Figure 3 of the main manuscript.*

| Participant | | Movement/Field range: Min – Max (Range) |
|---|---|---|

| | Area Covered ($m^2$) | Left-Right (m) | Forward-Back (m) | Field Change (single channel) (nT) |
|---|---|---|---|---|
| 1 | 1.59 | -1.12 – 0.31 (1.43) | -0.58 – 0.94 (1.52) | -3.74 – 5.09 (8.83) |
| 2.1 | 2.46 | -0.61 – 0.55 (1.16) | -1.36 – 1.28 (2.64) | -3.38 – 3.75 (7.14) |
| 2.2 | 6.03 | -1.48 – 1.39 (2.87) | -1.64 – 1.66 (3.30) | -11.91 – 7.21 (19.12) |
| 3 | 3.50 | -1.03 – 1.15 (2.18) | -1.29 – 1.27 (2.56) | -6.35 – 6.25 (12.61) |

*Supplementary Table 1. Range of movement and magnetic field for each open-loop walking recording.*

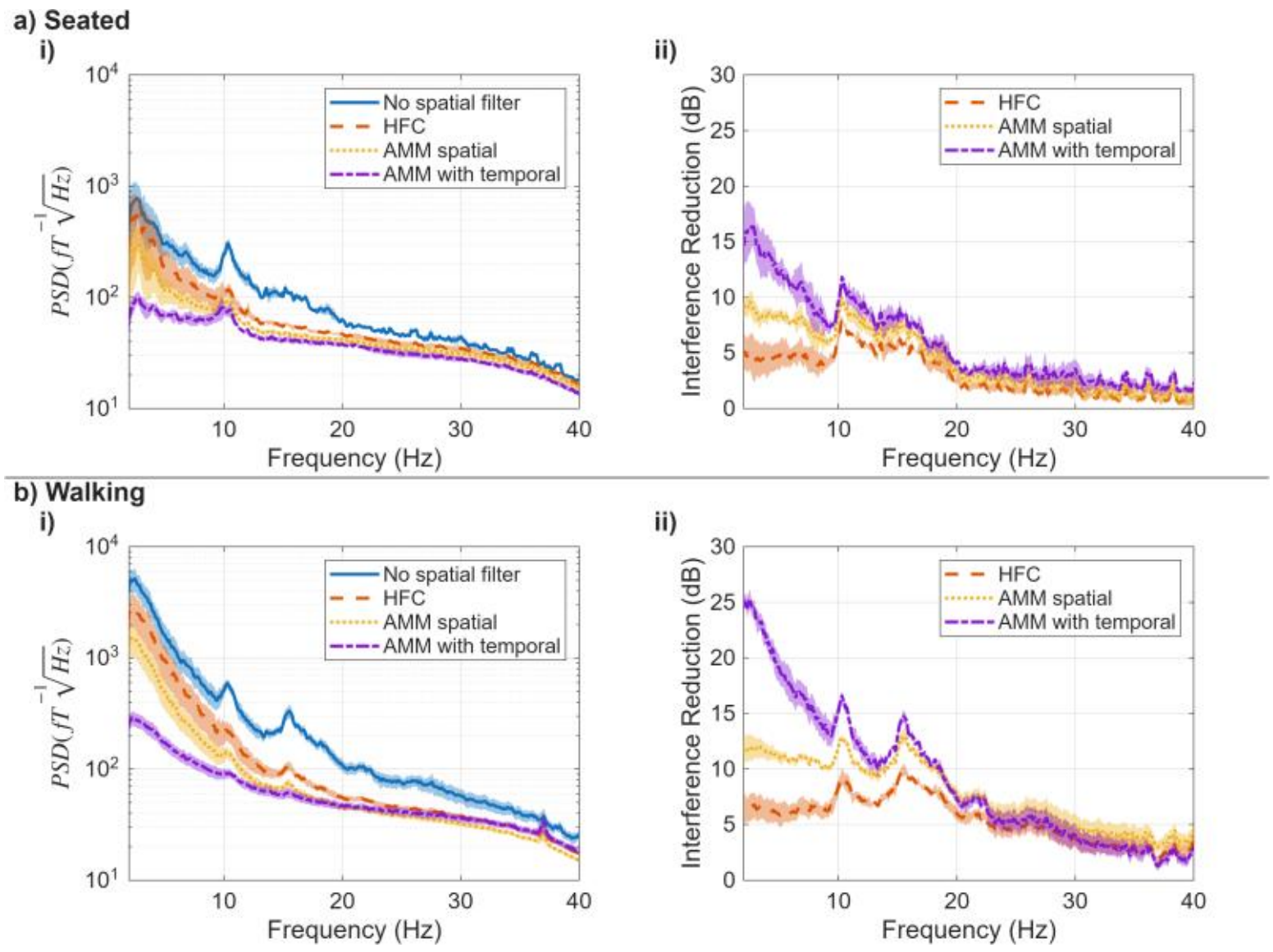


*Supplementary Figure 11. Power spectral density (PSD) (i) and interference reduction (ii) from post-hoc application of HFC and AMM to the open-loop data. Mean PSD curves and interference reduction factors were calculated over channels for each dataset separately and then averaged; the width of the curve shown here is given by the standard error of the mean across datasets. Data were pre-filtered to between 2 Hz and 40 Hz and so any performance outside of this range should be disregarded.*

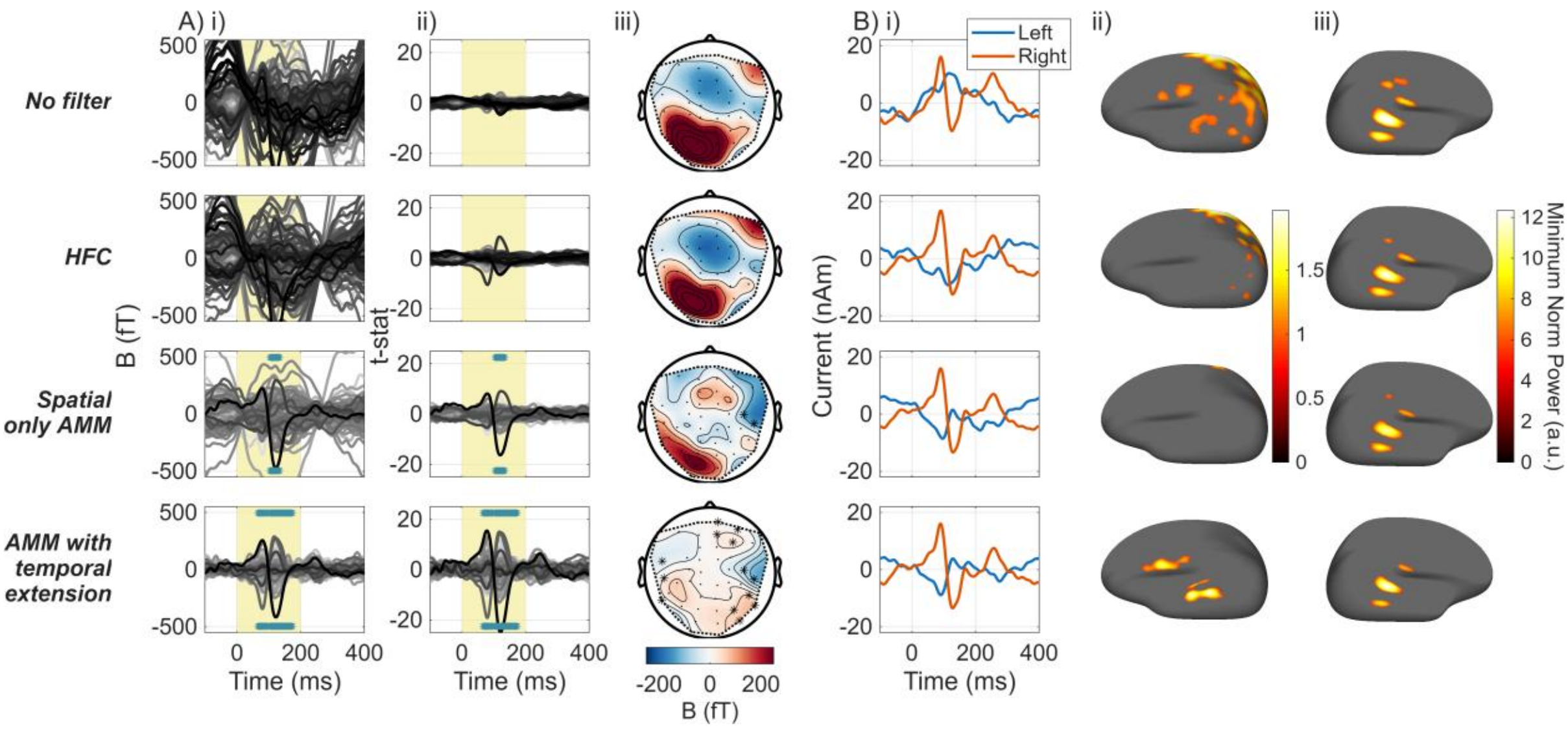


*Supplementary Figure 12. Auditory evoked responses for participant 1 while seated with the OPMs under open-loop operation, shown after different spatial filters. See Figure 5 of the main manuscript for full caption.*

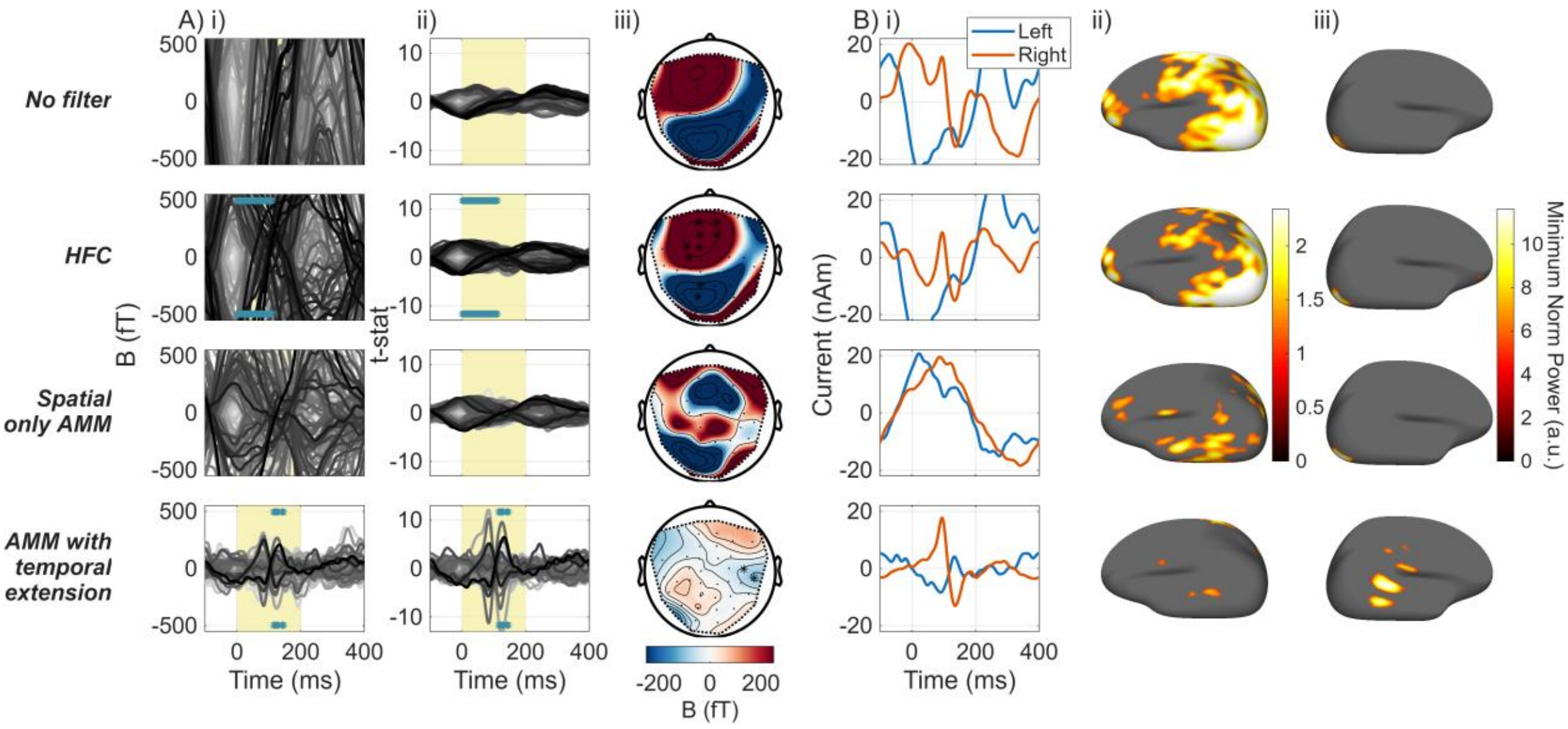


*Supplementary Figure 13. Auditory evoked responses for participant 1 while walking with the OPMs under open-loop operation, shown after different spatial filters. See Figure 5 of the main manuscript for full caption.*

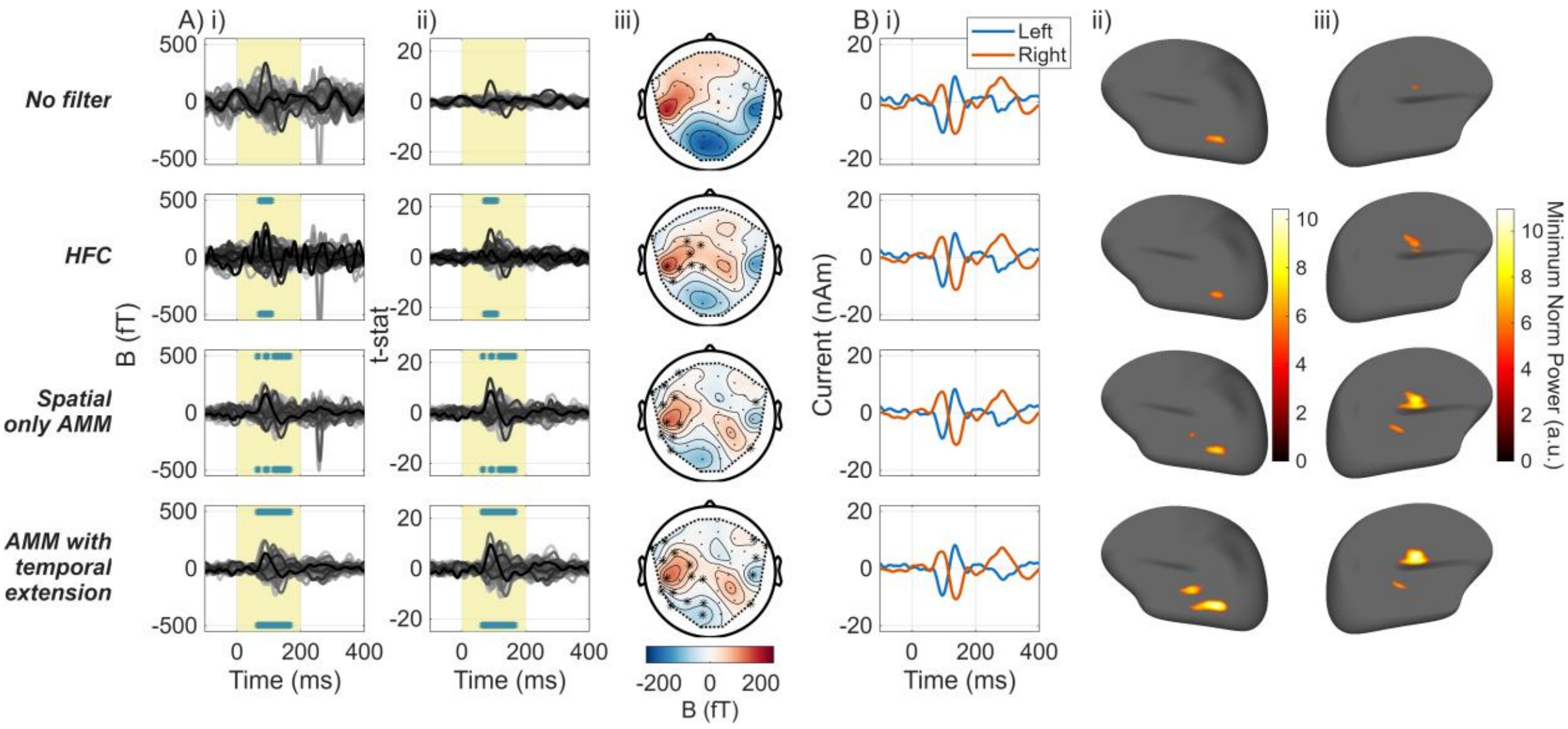


*Supplementary Figure 14. Auditory evoked responses for participant 2 while seated with the OPMs under open-loop operation, shown after different spatial filters. See Figure 5 of the main manuscript for full caption.*

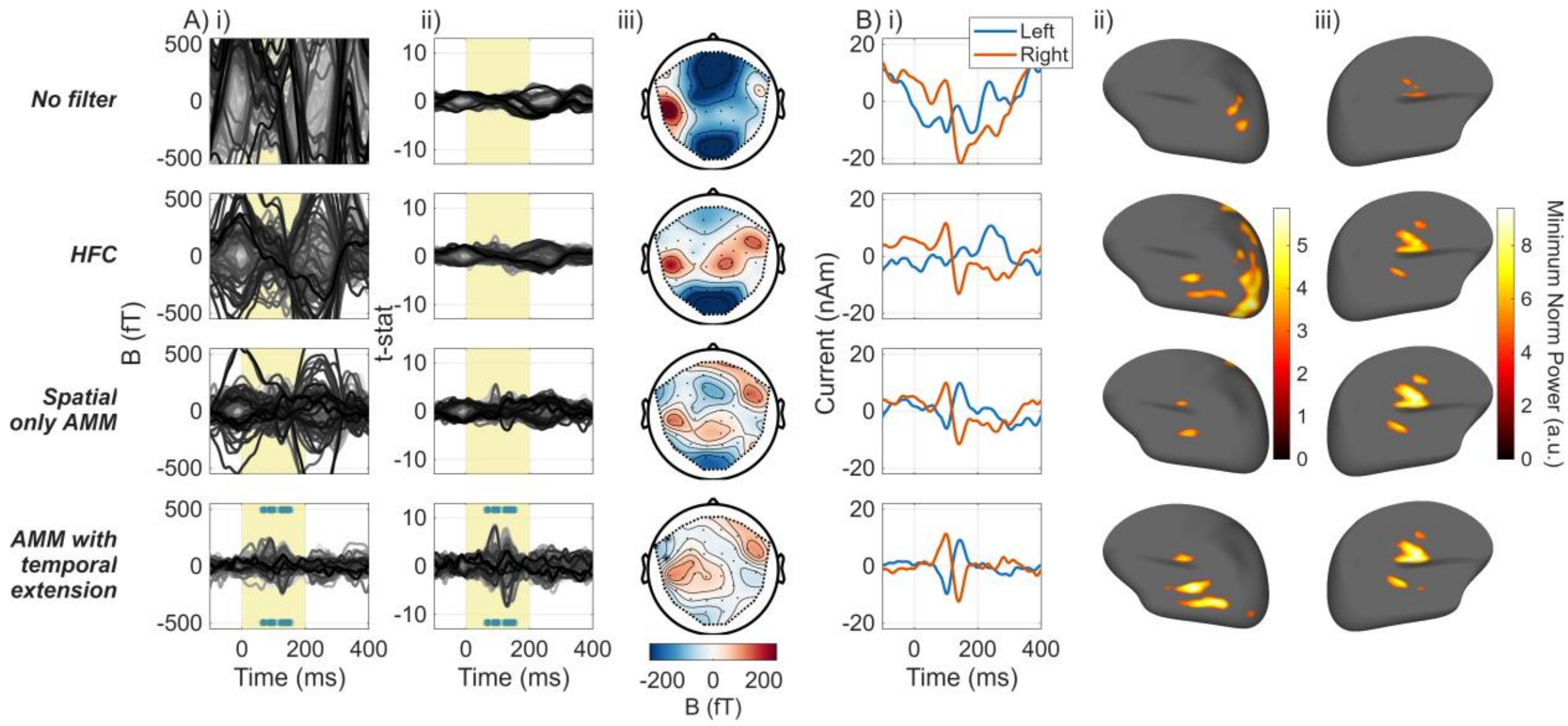


*Supplementary Figure 15. Auditory evoked responses for participant 2 while walking recording 2.1 with the OPMs under open-loop operation, shown after different spatial filters. See Figure 5 of the main manuscript for full caption.*

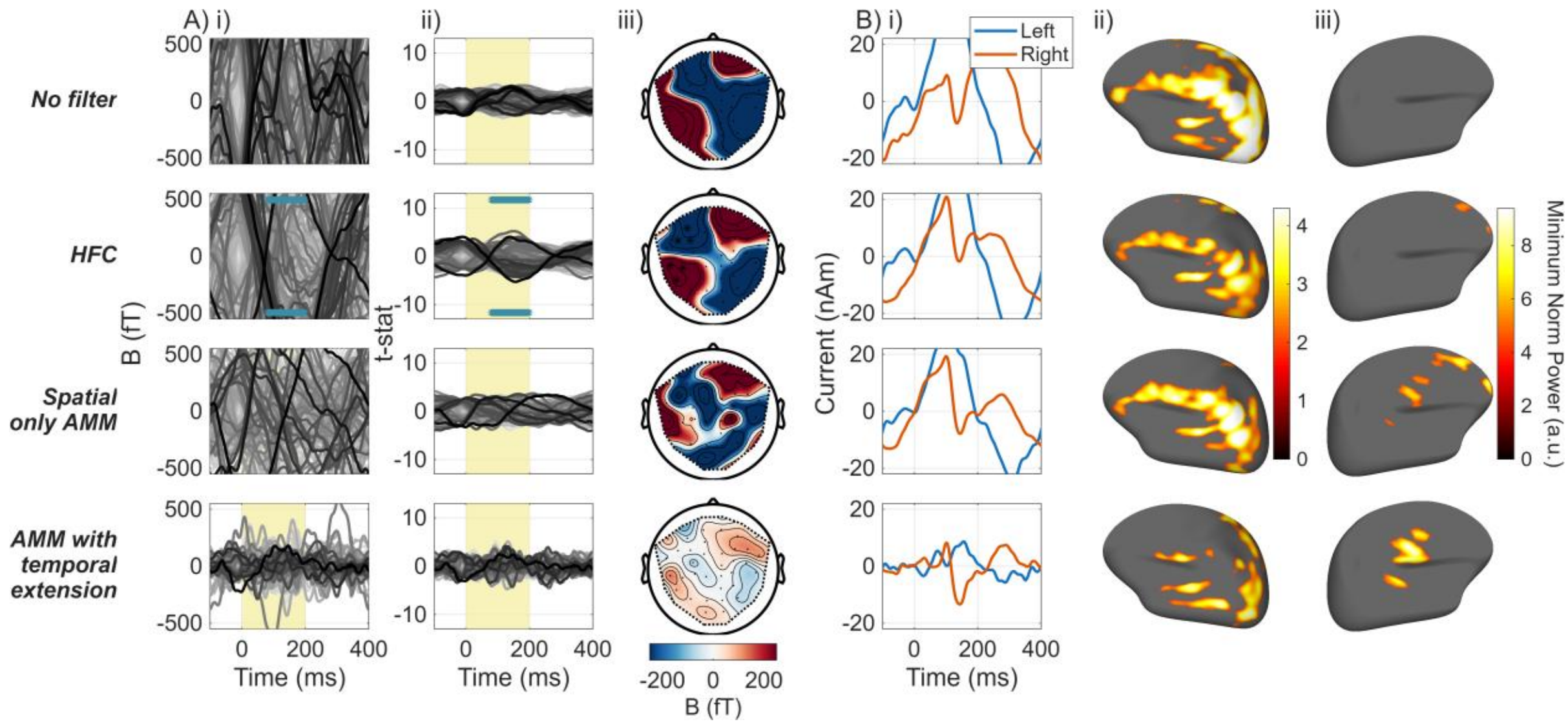


*Supplementary Figure 16. Auditory evoked responses for participant 2 while walking recording 2.2 with the OPMs under open-loop operation, shown after different spatial filters. See Figure 5 of the main manuscript for full caption.*

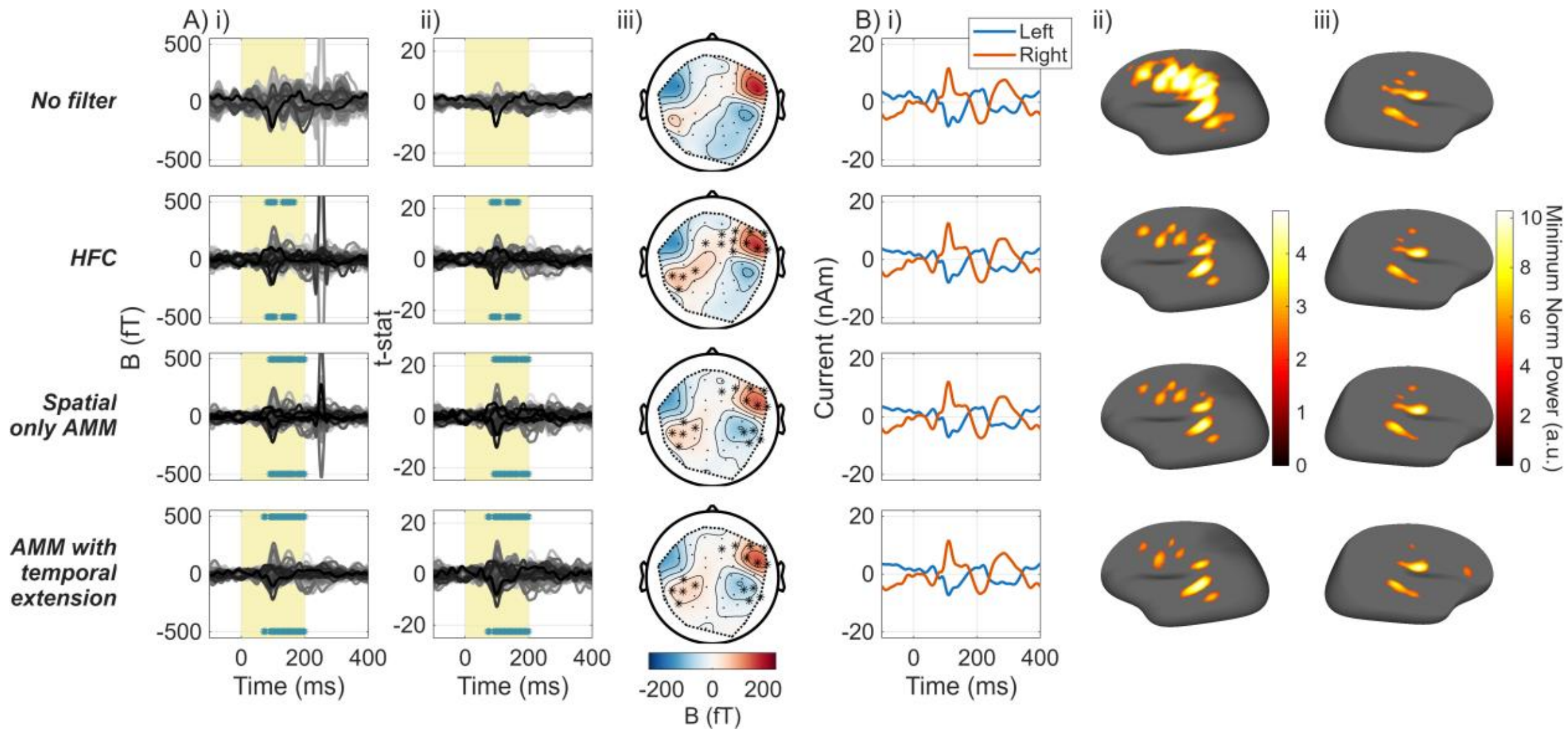


*Supplementary Figure 17. Auditory evoked responses for participant 3 while seated with the OPMs under open-loop operation, shown after different spatial filters. See Figure 5 of the main manuscript for full caption.*

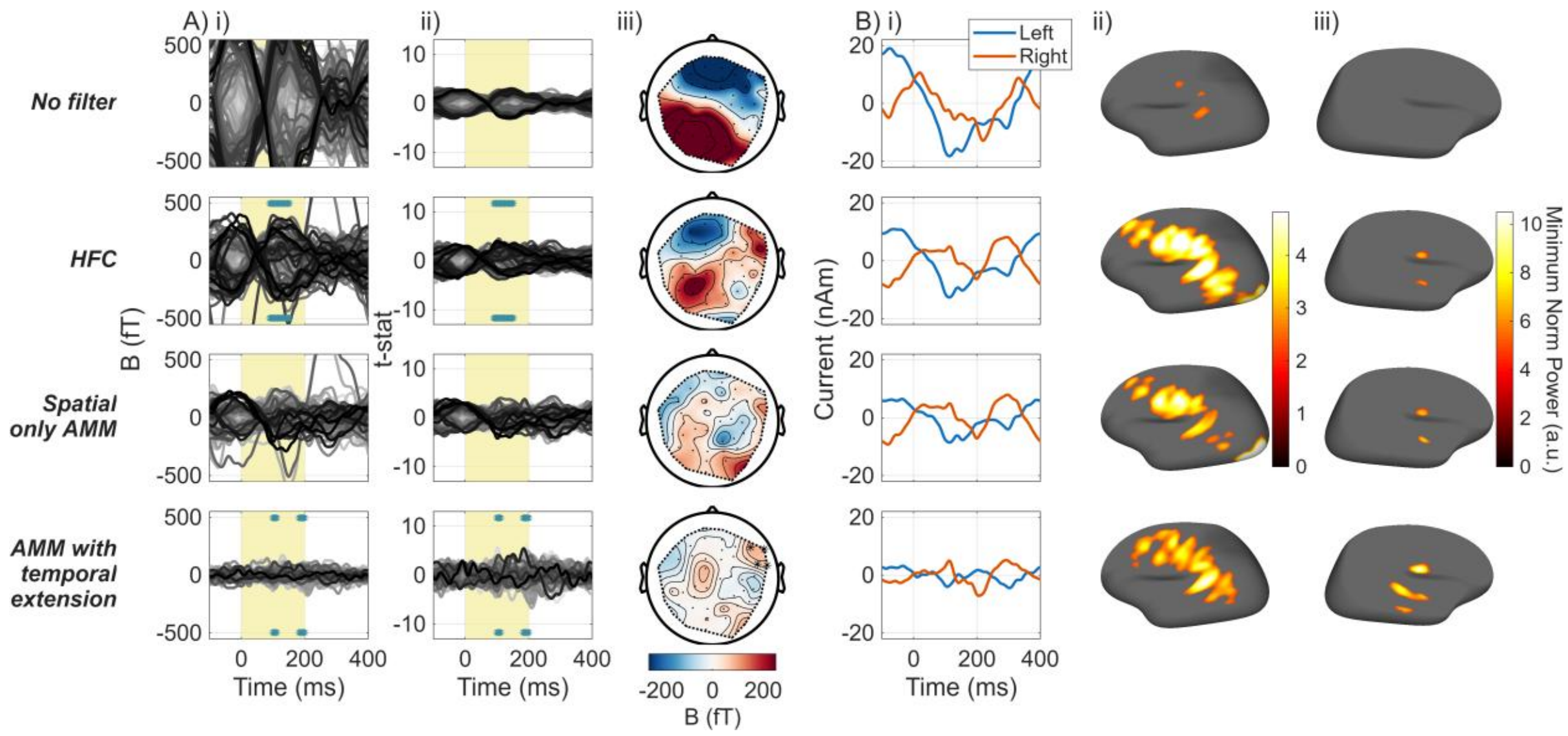


*Supplementary Figure 18. Auditory evoked responses for participant 3 while walking with the OPMs under open-loop operation, shown after different spatial filters. See Figure 5 of the main manuscript for full caption.*

# 6 Movement Speed

An estimate of the speed of each participant, both in traversing the space and in the corresponding field change, is given in Supplementary Figure 19 for the closed loop recordings and Supplementary Figure 20 for open loop recordings. The rate of change of the magnetic field is calculated by calculating difference between consecutive datapoints and dividing by the time difference between datapoints. This is calculated for each channel separately and then the data aggregated to plot the histograms. The velocity of the participant throughout the space is estimated only during continuous data segments, i.e. where head position was successfully recorded without occlusion and interpolation of marker position was only performed in Motive. During these data segments, the difference in position between consecutive datapoints was divided by the time difference between datapoints.

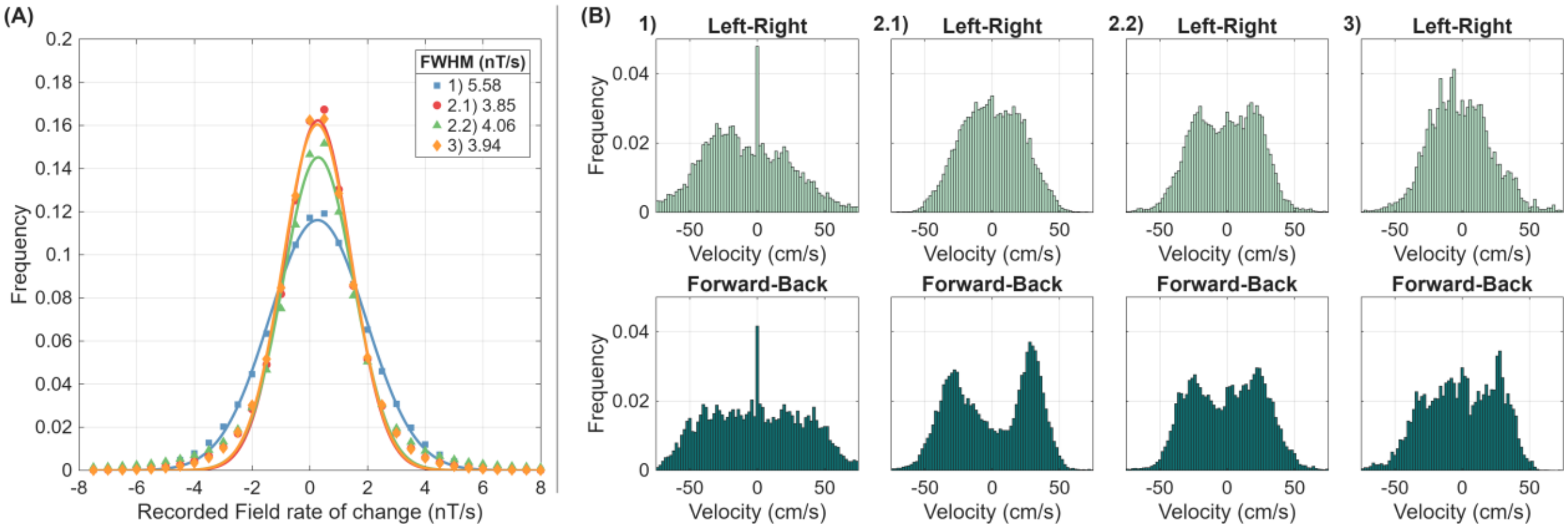


*Supplementary Figure 19. Histograms of instantaneous rate of change of magnetic field (panel (A)) and position (panel (B)) for each closed loop walking dataset (participants 1–3). The rate of change of magnetic field (aggregated over sensors) has been fitted with a Gaussian curve with the Full Width Half Maximum given in the legend.*

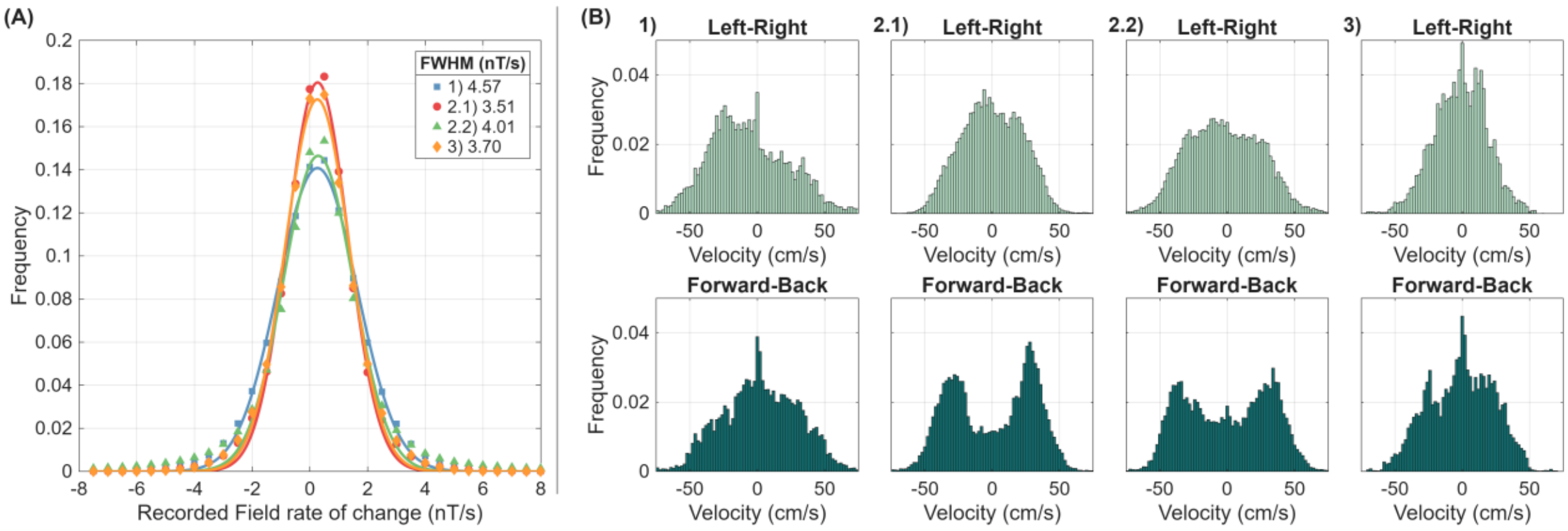


*Supplementary Figure 20. Histograms of instantaneous rate of change of magnetic field (panel (A)) and position (panel (B)) for each open loop walking dataset (participants 1–3). The rate of change of magnetic field (aggregated over sensors) has been fitted with a Gaussian curve with the Full Width Half Maximum given in the legend.*
